# Stable Radical Cations of *N*,*N′*‐Diarylated Dihydrodiazapentacenes

**DOI:** 10.1002/chem.201904308

**Published:** 2019-12-16

**Authors:** Gaozhan Xie, Victor Brosius, Jie Han, Frank Rominger, Andreas Dreuw, Jan Freudenberg, Uwe H. F. Bunz

**Affiliations:** ^1^ Organisch-Chemisches Institut Ruprecht-Karls-Universität Heidelberg Im Neuenheimer Feld 270 69120 Heidelberg Germany; ^2^ Interdisziplinäres Zentrum für Wissenschaftliches Rechnen and Physikalisch-Chemisches Institut Ruprecht-Karls-Universität Heidelberg Im Neuenheimer Feld 205 69120 Heidelberg Germany; ^3^ InnovationLab Speyerer Str. 4 69115 Heidelberg Germany; ^4^ Centre for Advanced Materials Ruprecht-Karls-Universität Heidelberg Im Neuenheimer Feld 225 69120 Heidelberg Germany

**Keywords:** *N*,*N′*-diaryldiazapentacenes, oxidation, radical cation, single crystal structure

## Abstract

A series of quinoidal *N*,*N′*‐diaryldiaza‐*N*,*N′*‐dihydropentacenes (Quino) was prepared in a two‐step reaction, starting from quinacridone. Oxidation of Quino furnishes stable radical cations, isoelectronic to the radical anions of the azaacenes, whereas the dicationic species are isoelectronic to neutral azapentacenes. The spectroscopic properties of the diaryldiazapentacenes and their oxidized mono‐ and dications are equivalent to that of the dianion of tetraazapentacene (TAP), its radical anion and the neutral TAP.

## Graphical abstract

**Radicalized**: A series of quinoidal *N*,*N*′‐5,7,12,14‐tetraaryl‐5,12‐dihydropentacenes were oxidized into their radical cations and dications, the latter electronically resembling highly acceptor‐substituted diazapentacenes.

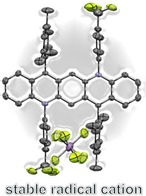

Herein, we describe the synthesis and characterization of novel *N*,*N′*‐diaryldiazapentacenes in their neutral, radical cation, and closed‐shell dication states, spectroscopically similar to the dianion, radical anion, and neutral tetraazapentacenes.^1^

Acenes and *N*‐heteroacenes^2^, ^3^ play a fundamental role in chemistry, in material science, and organic electronics, particularly as charge transport materials.^4^ Larger *N*‐heteroacenes are easily reduced into their *N*,*N′*‐dihydro compounds, much longer known and more stable than the *N*‐acenes themselves. There are only a few reports on the chemistry of the reduced compounds from Miao,^5^ Beckert,^6^ and Koutentis,^7^ and surprisingly little is known for the redox chemistry of these materials.

Chi et al. spectacularly investigated sulfur‐ and oxygen‐embedded quinodimethane acene analogues.^8^ They demonstrated that their dications display properties very similar to those of the acenes of similar length, but for the larger representatives these dications are—contrary to the isoelectronic acenes themselves—isolable, stable, and can be characterized. In this contribution, we extend this concept to *N*‐heterocycles.

Starting from quinacridone, copper‐catalyzed *N*‐arylation with different 4‐iodoarenes gives QA‐*t*Bu, ‐OMe, and ‐CF_3_ in excellent yields. A double nucleophilic addition reaction of the organolithium compound formed from bromomesitylene and BuLi followed by treatment with SnCl_2_ in THF furnishes the three Quino‐structures in 32–39 % yield (Scheme 1). Oxidation with AgSbF_6_ (1 equiv) gives the monocations Quino^+.^ displaying EPR spectra without fine‐structure (*g*_e_=2.0017, see Figure S1 in the Supporting Information). To obtain the dications Quino^2+^, the stronger oxidant NO^+^PF_6_^−^ is employed. All three neutral Quino compounds are stable in the solid state. In dichloromethane (DCM), under ambient conditions, the electron‐rich Quino‐OMe is the least stable (decomposition after 3 h, Figure S2), while Quino‐CF_3_ is much more persistent (≈20 % absorption intensity loss after 9 h). Surprisingly, Quino^+.^ species are stable and their absorption spectra (Figure S3) in DCM remain unchanged under ambient conditions for at least 24 h.

**Scheme 1:**
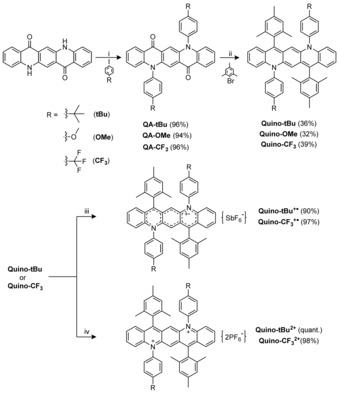
Synthetic details towards nitrogen‐embedded quinoidal pentacenes and their corresponding radical cations and dications. i) 2,2,6,6‐Tetramethyl‐3,5‐heptanedione, K_2_CO_3_, CuI, DMF, 146 °C, 36 h; ii) (a) *n*BuLi, THF, −78 °C to room temperature (r.t.), 12 h; (b) SnCl_2_, THF, r.t. 1 h; iii) AgSbF_6_, DCM, r.t., 12 h; iv) NO^+^PF_6_^−^, DCM, r.t., 12 h.

Figure 1 displays the UV/Vis spectra of the three oxidation states of Quino‐*t*Bu and Quino‐CF_3_. They are very similar, as expected. The neutral Quino‐*t*Bu displays an absorption spectrum with a maximum at 574 nm. Upon oxidation into the radical cation a large redshift to 1026 nm is observed. The dication on the other hand displays an absorption spectrum with a *λ*_max_ of 672 nm. These spectra resemble very much the UV/Vis spectra that are observed for azaacenes but in the sequence dianion–radical anion–neutral species (cf. absorption maxima sketched for TAP in Figure 1, top). Apparently, these species are isoelectronic (see Figure 2; Quino/TAP^2−^ 24 e−, Quino^+.^/TAP^−.^ 23 e−, Quino^2+^/TAP 22 e− per backbone). A direct comparison to pentacene in different oxidation states is hindered by solvent effects. A blueshift is reported when comparing the dication to the monocation of pentacene.^9^

**Figure 1:**
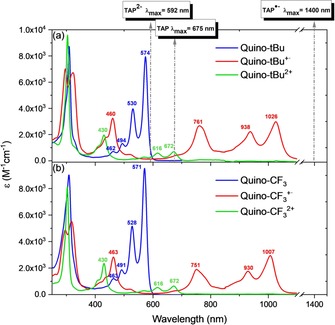
Absorption spectra of a) Quino‐*t*Bu, Quino‐*t*Bu^+.^, and Quino‐*t*Bu^2+^ (dotted arrows: maximum absorptions of TAP, TAP^−.^, and TAP^2−^); b) Quino‐CF_3_, Quino‐CF_3_^+.^, and Quino‐CF_3_^2+^ measured in dichloromethane.

**Figure 2:**
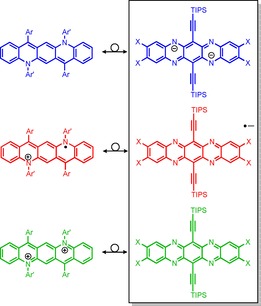
Comparison of the electronic properties of Quino and of TAP. X=H, Br.

To rationalize the experimental results, we performed quantum chemical calculations of the vertical excited states at TD‐DFT/B3LYP/6‐311G** level of theory. As can be seen from Figure S5, the simulated absorption spectra of the Quino‐CF_3_ species are consistent with the experiment. The lowest electronic transition of Quino‐CF_3_ is a HOMO–LUMO transition with an excitation energy of 2.22 eV located at 559 nm in the simulated absorption spectrum. Due to neglect of vibrational effects, the broadening of the absorption bands is not reproduced. The lowest absorption band of Quino‐CF_3_^+.^ is redshifted to the near‐infrared region with contributions from two electronic transitions of HOMOα–LUMOα and HOMOβ–LUMOβ characters. Furthermore, the first bright state of Quino‐CF_3_^2+^ lies in between those of Quino‐CF_3_ and Quino‐CF_3_^+.^ with a *λ*_max_ of 653 nm. Therefore, the first absorption band of Quino‐CF_3_ is blueshifted compared to its cationic and dicationic species. A similar blueshift feature also exhibits in dianionic TAPs^2−^, when compared with TAP and TAP^−.^.1b Hence, the character of each vertical transition for Quino‐CF_3_ and TAP is compared and shown in Table S2. It can be easily found that Quino/TAP^2−^, Quino^+.^/TAP^−.^ and Quino^2+^/TAP share common transition characters. For example, the most important contribution to the first bright state (BS1) of Quino‐CF_3_ is a HOMO to LUMO transition, the BS1 of TAP^2−^ is analogously also mainly a HOMO–LUMO transition with similar molecular orbital shapes. Thus, the resemblance of the peak position in the absorption spectra and the similar characteristics of the electronic transitions corroborate the isoelectronic properties of Quino/TAP^2−^, Quino^+.^/TAP^−.^ and Quino^2+^/TAP.

The Quino‐compounds are easily and reversibly oxidized at very similar potentials (Figure S7). For Quino‐CF_3_, the first oxidation potential to the radical cation is located at −0.11 V and the second one to the dication at +0.51 V (both vs. Fc/Fc^+^). The more interesting experiment is the consecutive cyclovoltammetric reduction of the dications (example of Quino‐CF_3_^2+^, Figure S9). The first reduction potential of Quino‐CF_3_^2+^ is at −0.28 V and the second one is at −0.83 V vs. Fc/Fc^+^. This reduction series is analogous to the reduction of TAP into the TAP radical anion at −0.79 V and the second step is analogous to the reduction of the TAP‐radical anion into the dianion (−1.23 V). As expected, the Quino^2+^ species are more electron accepting than the neutral, in the case of the CF_3_‐species about 0.51 V. The second reduction is easier for the Quino series than for TAP, even if one starts from the radical cation→neutral compound and for TAP from the radical anion→dianion.

This facile oxidation is testament to the formation of an aromatic system, that is, Quino‐CF_3_^2+^. To shed further light on this issue we performed quantum chemical NICS‐calculations (Figure 3). In the neutral Quino‐CF_3_, NICS‐values of the three interior rings are positive with the maximum value up to +9.5, yet smaller than those reported for the formally antiaromatic ring in *N*,*N′*‐dihydrotetraazapentacene (+23, NICS (0)_zz_).^10^ Upon monooxidation, the overall aromaticity of the open‐shell system, *as calculated by NICS*, increases, and all of the rings now display negative NICS‐values, with the outer ones and the middle one being more aromatic than the formal pyridine‐like ones. Further oxidation to the closed‐shell Quino‐CF_3_^2+^, necessitating a stronger oxidant preparatively, also results in a fully aromatic system with similarly negative NICS values.

**Figure 3:**
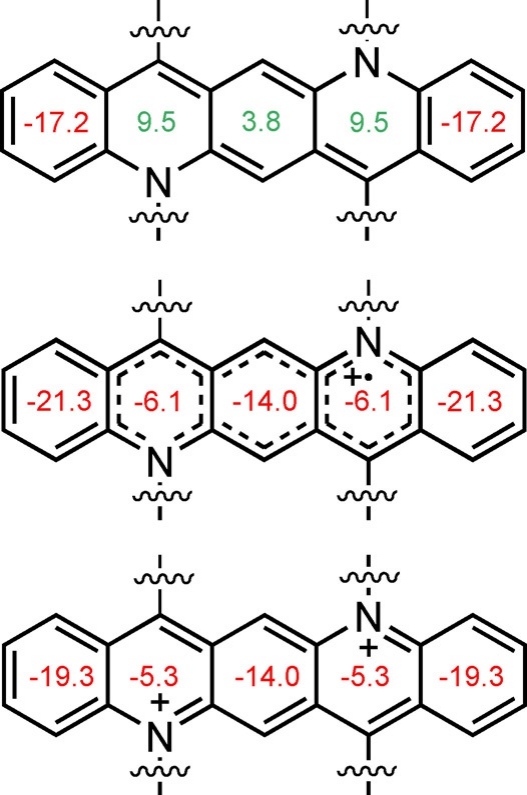
NICS(1)_zz_ values of Quino‐CF_3_ (top), Quino‐CF_3_^+.^ (middle), and Quino‐CF_3_^2+^ (bottom) calculated at B3LYP/6‐311G** level employing a PCM model for DCM solution.

We obtained a single crystalline specimen of Quino‐CF_3_ and its radical cation by slow evaporation of THF and acetone, respectively. Figure 4 displays the single crystal structure and the bond distances of the neutral and the radical cation of Quino‐CF_3_. The neutral specimen displays a significant bond alternation, in accordance with the DFT optimized geometry, that strongly suggests a quinoidal structure, as expected from the simple resonance structures.^11^ The quinoidal character decreases when going from the neutral compound to the radical cation, as expected. In the calculated structures (Figure 4) this is also observed.

**Figure 4:**
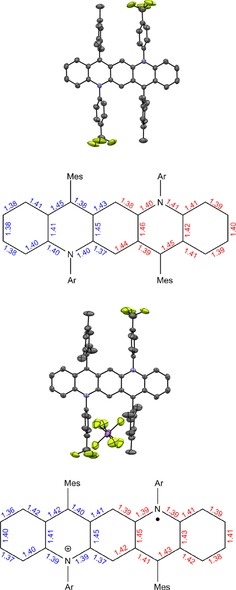
Single‐crystal structures and bond lengths (blue: derived from the crystal structure; red: calculated at the DFT/B3LYP/6‐31+G** level) of neutral Quino‐CF_3_ compound (top) and its radical cation (bottom).

The structural spectroscopic and quantum chemical data for the Quinos and their radical cations and dications display a significant resemblance to the data collected for the series of TAP dianion, radical anion, and TAP as neutral compound. The resemblance is most striking if one looks at the spectroscopic data, and while the TAP dianion does not feature a distinct quinoidal structure, Quino does so. Yet the spectra are very similar. More remarkable is that the TAP radical anion and the Quino radical cations display red shifted spectra similar to each other suggesting an extensive isoelectronic relationship (Figure 2). Over all, we have prepared neutral, *N*,*N′*‐diaryldiazapentacenes. These species are electronically equivalent to diazapentacene dianions. They are easily oxidized and the radical cation is environmentally stable. In the future we will prepare *N*,*N*′‐diaryldiazapentacenes with tailored packing behavior that should function as attractive “Ersatz”‐tetraazapentacenes.^12^

## Experimental Section

**7,14‐Dimesityl‐5,12‐dihydro‐5,12‐bis(4‐trifluoromethylphenyl) diazapentacene (Quino‐CF_3_)**: 2‐Bromomesitylene (552 mg, 0.42 mL, 2.77 mmol, 8.00 equiv) was dissolved in dry THF (20 mL) under protection of N_2_. *n*BuLi (0.83 mL, 2.08 mmol, 2.5 m, 6.00 equiv) was added dropwise at −78 °C. Three hours later, QA‐CF_3_ (210 mg, 0.35 mmol, 1.00 equiv) was added and the temperature was allowed to increase to ambient. After stirring for another 12 h, the reaction was quenched by water (1 mL), and SnCl_2_ (3.61 g, 19.0 mmol, 50 equiv) was added into the flask. After that, the mixture was stirred at ambient temperature for 1 h. The mixture was poured into ethanol (100 mL), furnishing a red precipitate. It was collected by filtration and washed with water and ethanol. Yield: 110 mg, 0.14 mmol, 39 %. Mp: >400 °C (decomposition). ^1^H NMR ([D_8_]THF, 600 MHz, 295.1 K): *δ*=7.84–7.78 (m, 4 H), 7.43–7.36 (m, 4 H), 6.77–6.71 (m, 4 H), 6.60–6.52 (m, 2 H), 6.48–6.41 (m, 2 H), 6.27–6.18 (m, 2 H), 5.89–5.81 (m, 2 H), 3.89–3.79 (m, 2 H), 2.24–2.21 (s, 6 H), 1.96–1.93 (s, 12 H) ppm. ^13^C NMR ([D_8_]THF, 151 MHz, 22.0 °C): *δ*=144.2, 144.1, 141.0, 137.4, 133.1, 131.9, 131.4, 131.2, 131.0, 129.0, 128.9, 126.8, 126.6, 126.0, 124.2, 123.8, 122.3, 113.8, 96.7, 21.0, 19.8 ppm. IR: *ṽ*=3423, 3035, 2911, 1712, 1592, 1407, 1197, 973, 914, 821, 757, 727, 605, 566 cm^−1^. HRMS (MALDI) *m*/*z*: [*M*]^+^: calcd for C_52_H_40_F_6_N_2_: 806.3096; found 806.3099, correct isotope distribution.

Quino‐CF_3_^+.^: In the glovebox under N_2_, to a stirring solution of Quino‐CF_3_ (50.6 mg, 63.9 μmol, 1.00 equiv) in 10 mL DCM, AgSbF_6_ (22.0 mg, 63.9 μmol, 1.00 equiv) in 1 mL CH_3_CN solution was added dropwise and the reaction mixture was stirred for 12 h at r.t. After that, the mixture was filtered and the solvent was removed under reduced pressure to give radical cation as a dark brown solid. Yield: 64.6 mg, 62.0 μmol, 97 %. IR: *ṽ*=3065, 2924, 2852, 1560, 1316, 1248, 1062, 1160, 746, 643 cm^−1^.

Quino‐CF_3_^2+^: In the glovebox under N_2_, to a stirring solution of Quino‐CF_3_ (50.6 mg, 63.9 μmol, 1.00 equiv) in 10 mL DCM, NO^+^PF_6_^−^ (22.3 mg, 128 μmol, 2.00 equiv) in 1 mL CH_3_CN solution was added dropwise and the reaction mixture was stirred for 12 h at r.t. After that, the mixture was filtered and the solvent was removed under reduced pressure to give the dication as a deep green solid. Yield: 68.7 mg, 62.6 μmol, 98 %. ^1^H NMR ([D_3_]acetonitrile, 600 MHz, 295.1 K): *δ*=8.40–8.32 (m, 2 H), 8.20–8.11 (m, 4 H), 8.07–8.01 (m, 2 H), 7.94–7.83 (m, 8 H), 7.79–7.72 (m, 2 H), 7.13 (s, 4 H), 2.45–2.42 (m, 6 H), 1.76–1.71 (m, 12 H) ppm. ^13^C NMR ([D_3_]acetonitrile, 151 MHz, 295.1 K): *δ*=168.5, 147.5, 145.7, 142.8, 140.9, 137.8, 137.7, 134.9, 131.4, 131.2, 130.5, 130.0, 129.6, 129.4, 127.7, 125.8, 124.0, 123.2, 121.6, 21.5, 20.6 ppm. IR: *ṽ*=3095, 2920, 2852, 1316, 1065, 830, 552 cm^−1^.

**Quantum chemical calculations**: The ground state geometries for the neutral, cationic, and dicationic of Quino‐CF_3_ were optimized at the B3LYP/6‐311G** level of theory, employing a polarizable continuum model using the integral equation formalism variant (IEFPCM)^13^ for DCM solvation. The optimized geometries were confirmed to be local minima (all frequencies are real). Upon these optimized geometries, additional calculations for nuclear‐independent chemical shift (NICS) values using gauge‐independent atomic orbitals (GIAO)^14^ as well as vertical excitations using time‐dependent density‐functional theory (TD‐DFT)^15^ were conducted at the same level of theory. All quantum chemical calculations were performed by using Gaussian 16 Rev. B.01.^16^

**Crystallographic data**: CCDC https://www.ccdc.cam.ac.uk/services/structures?id=doi:10.1002/chem.201904308 (Quino‐*t*Bu, Quino‐CF_3_, Quino‐CF_3_^+.^) contain the supplementary crystallographic data for this paper. These data are provided free of charge by http://www.ccdc.cam.ac.uk/.

## Conflict of interest

The authors declare no conflict of interest.

## Supporting information

As a service to our authors and readers, this journal provides supporting information supplied by the authors. Such materials are peer reviewed and may be re‐organized for online delivery, but are not copy‐edited or typeset. Technical support issues arising from supporting information (other than missing files) should be addressed to the authors.

Supplementary
