# Supplementary material for: Stable Radical Cations of N,N′‐Diarylated Dihydrodiazapentacenes
Source: Chemistry. 2019 Dec 16;26(1):160–4. doi: 10.1002/chem.201904308 (PMC6973034; doi:10.1002/chem.201904308)
Supplement: Supplementary file 1 — Supplementary [file CHEM-26-160-s001.pdf]

CHEMISTRY
A European Journal

Supporting Information

Stable Radical Cations of *N,N′*-Diarylated Dihydrodiazapentacenes

Gaozhan Xie,[a] Victor Brosius,[a] Jie Han,[b] Frank Rominger,[a] Andreas Dreuw,*[b]
Jan Freudenberg,[a, c] and Uwe H. F. Bunz*[a, d]

chem_201904308_sm_miscellaneous_information.pdf

Index:

S1. Experimental Section 02

S2. Electron Spin Resonance Spectra 06

S3. Absorption and Emission Spectra 06

S4. Theoretical Studies 08

S5. Electrochemistry 11

S6. NMR Spectroscopy 13

S7. Mass Spectrometry 26

S8. Infrared Spectroscopy 29

S9. Crystal Structures 32

S10. References 35

S1. Experimental Section

Materials and Methods. Thin-layer chromatography (TLC) was carried out on Polygram SILG/UV254 plates from Macherey, Nagel&Co.KG (Düren, Germany) and examined under ultraviolet irradiation (254 and 365 nm). NMR spectra (^{1}H, ^{13}C) were recorded at Bruker Avance III 300, Bruker Avance III 400 or Bruker Avance III 600. Chemical shifts (δ) are given in parts per million (ppm) relative to internal solvent signals. The following abbreviations describe the signal multiplicities: s = single m = multiplet. IR spectra were recorded on a JASCO FT/IR-4100. High resolution mass spectra (HR-MS) were obtained from matrix-assisted laser desorption/ionization (MALDI) on a Bruker ApexQe hybrid 9.4 TFT-ICR-MS. Crystal structure analysis was accomplished on Bruker Smart CCD or Bruker APEX diffractometers.

Syntheses. All reagents were obtained from Sigma-Aldrich and ABCR without any further purification. Dimethyl formamide (DMF), tetrahydrofuran (THF), dichloromethane (DCM) and acetonitrile for reactions are dried before use. The radical cations and dications are synthesized in the glove box under N_2.

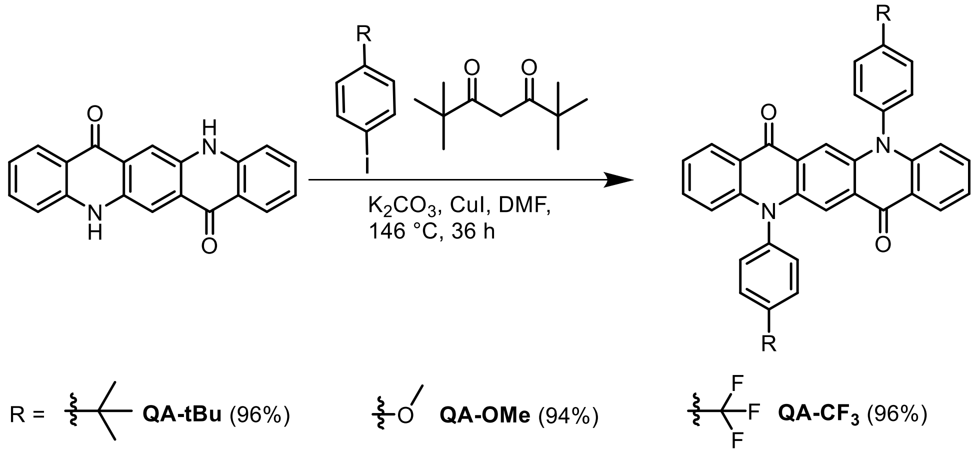

General procedure (**GP1**)：Quinacridone (200 mg, 0.64 mmol, 1 equivalent (eq.)), 4-iodoarenes (2.56 mmol, 4.00 eq), K_2CO_3 (354 mg, 2.56 mmol, 4.00 eq.), copper(I) iodide (48.8 mg, 0.26 mmol, 0.40 eq.), 2,2,6,6-tetramethyl-3,5-heptanedione (94.4 mg, 0.51 mmol, 0.80 eq.) and 20 ml DMF were added into a 100 ml flask under N_2. The mixture was stirred and heated to 146 °C for 36 h. After that, the mixture was allowed to cool to room temperature (r.t.) and an orange solid precipitated. The precipitate was collected by filtration and then washed by water and ethanol.

N,N'-Bis(4-tert-butylphenyl)quinacridone (**QA-tBu**)

4-*tert*-Butyliodobenzene (666 mg, 2.56 mmol, 4.00 eq) was used with **GP1** and **QA-tBu** was obtained as an orange powder. Yield: 354 mg, 0.62 mmol, 96%. Melting point (Mp): > 400 °C (decomposition). ^{1}H NMR (CD_2Cl_2, 400 MHz, 295 K): δ = 8.44-8.38 (m, 2H), 8.01-7.96 (m, 2H), 7.83-7.79 (m, 4H), 7.55-7.48 (m, 2H), 7.40-7.36 (m, 4H), 7.24-7.18 (m, 2H), 6.84-6.78 (m, 2H), 1.52 (s, 18H) ppm. ^{13}C {^{1}H} NMR (CD_2Cl_2, 100 MHz, 295 K): δ = 178.3, 153.7, 144.6, 138.5, 136.7, 134.3, 130.0, 128.9, 127.6, 126.1, 121.6, 121.2, 117.5, 115.4, 35.6, 31.8 ppm. IR: ṽ = 2953, 1630, 1602, 1481, 1435, 1286, 900, 755, 560, 490 cm^{-1}. MS (MALDI) m/z: [M]$^+$: calcd. for $C_{40}H_{36}N_2O_2$: 576.278; found 576.306; correct isotope distribution.

N,N'-Bis(4-methoxylphenyl)quinacridone (**QA-OMe**)

4-Methoxyliodobenzene (599 mg, 2.56 mmol, 4.00 eq) was used with (**GP1**) and **QA-OMe** was obtained as an orange powder. Yield: 316 mg, 0.60 mmol, 94%. Mp: > 400 °C (decomposition). ^{1}H NMR (CD_2Cl_2, 600 MHz, 295 K): δ = 8.45-8.37 (m, 2H), 8.00-7.96 (s, 2H), 7.56-7.51 (m, 2H), 7.38-7.35 (m, 4H), 7.31-7.27 (m, 4H), 7.25-7.18 (m, 2H), 6.89-6.82 (m, 2H), 4.03-3.98 (s, 6H) ppm. ^{13}C {^{1}H} NMR (CD_2Cl_2, 151 MHz, 295 K): δ = 178.3, 161.0, 144.7, 138.6, 134.3, 131.8, 131.6, 127.5, 126.0, 121.5, 121.1, 117.4, 116.9, 115.3, 56.3 ppm. IR: ṽ = 2962, 2913, 2863, 1525, 1312, 738, 571 cm^{-1}. MS (MALDI) m/z: [M]$^+$: calcd. for $C_{34}H_{24}N_2O_4$: 524.576; found 524.204; correct isotope distribution.

N,N'-Bis(4-trifluoromethylphenyl)quinacridone (**QA-CF_3**)

4-Trifluoromethyliodobenzene (697 mg, 2.56 mmol, 4.00 eq) was used with (**GP1**) and **QA-CF_3** was obtained as an orange powder. Yield: 370 mg, 0.62 mmol, 96%. Mp: > 400 °C (decomposition). ^{1}H NMR (CD_2Cl_2, 400 MHz, 295 K): δ = 8.40-8.29 (m, 2H), 8.13-8.04 (m, 4H), 7.91-7.83 (s, 2H), 7.70-7.64 (m, 4H), 7.57-7.51 (m, 2H), 7.27-7.17 (m, 2H), 6.78-6.67 (m, 2H) ppm. ^{13}C {^{1}H} NMR (CD_2Cl_2, 151 MHz, 295 K): δ = 177.9, 143.9, 142.8, 138.0, 134.7, 131.7, 129.3, 127.8, 126.0, 122.2, 121.2, 117.0, 115.1 ppm. IR: ṽ = 3084, 2943, 1609, 1483, 1320, 1096, 1062, 754, 609 cm^{-1}. HRMS (MALDI) m/z: [M]$^+$: calcd. for $C_{34}H_{18}F_6N_2O_2$: 600.5204; found 601.1353; correct isotope distribution.

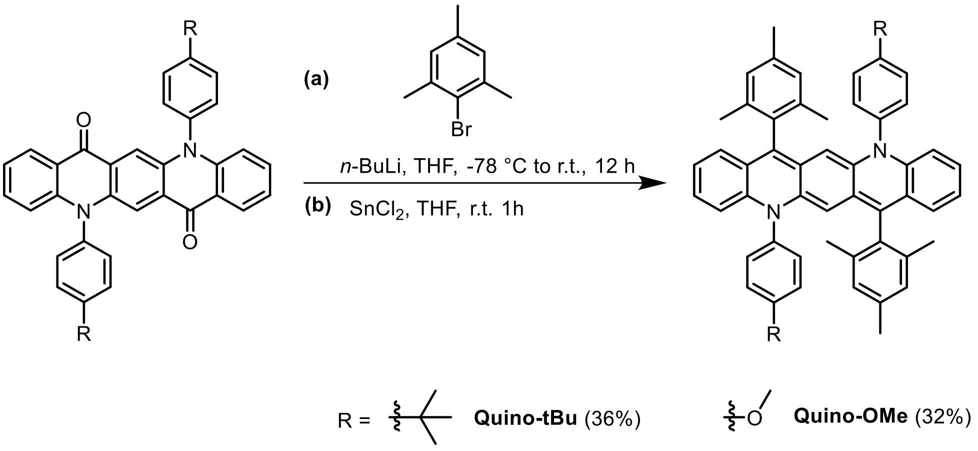

General procedure (**GP2**): 2-Bromomesitylene (552 mg, 0.42 mL, 2.77 mmol, 8.00 eq.) was dissolved in 20 ml dry THF under protection of N_2. *n*-BuLi (0.83 mL, 2.08 mmol, 2.5 M, 6.00 eq.) was added dropwise at -78 °C. After 3 h, N,N'-diphenylquinacridone derivatives (0.35 mmol, 1.00 eq.) were added and the temperature was allowed to increase to r.t.. After stirring for another 12 h, the reaction was quenched by 1 ml water followed by adding $SnCl_2$ (3.61 g, 19.0 mmol, 50 eq.) into the flask directly. After that, the mixture was stirred at r.t. for 1 h. The mixture was poured in 100 mL ethanol and the red precipitate appeared. The precipitate was collected by filtration and washed by water and ethanol.

7,14-Dimesityl-5,12-dihydro-5,12-bis(4-tert-butylphenyl)diazapentacene (**Quino-tBu**)

QA-tBu (200 mg, 0.35 mmol, 1.00 eq) was used with **GP2** and **Quino-tBu** was obtained as a red powder. Yield: 97.9 mg, 0.13 mmol, 36%. Mp: > 400 °C (decomposition). ^1H NMR (THF-d_8, 600 MHz, 295 K): δ = 7.55-7.47 (m, 4H), 7.06-7.03 (m, 4H), 6.75-6.72 (s, 4H), 6.52-6.47 (m, 2H), 7.37-6.32 (m, 2H), 6.07-6.02 (m, 2H), 5.88-5.83 (m, 2H), 4.15-4.10 (s, 2H), 2.24-2.20 (s, 6H), 1.99-1.93 (s, 12H), 1.38-1.34 (s, 18H) ppm. ^{13}C {^1H} NMR (THF-d_8, 150 MHz, 295 K): δ = 151.9, 144.2, 141.5, 137.7, 137.4, 136.3, 133.4, 131.6, 129.6, 128.7, 128.2, 126.5, 126.0, 123.4, 122.7, 121.4, 113.8, 35.1, 31.6, 21.3, 19.7 ppm. IR: $\tilde{v}$ = 2962, 2913, 2863, 1525, 1312, 738, 571 cm^{-1}. HRMS (MALDI) m/z: $[M]^+$: calcd. for $C_{58}H_{58}N_2$: 782.4600; found 782.4604; correct isotope distribution.

7,14-Dimesityl-5,12-dihydro-5,12-bis(4-methoxylphenyl)diazapentacene (**Quino-OMe**)

QA-OMe (184 mg, 0.35 mmol, 1.00 eq) was used with **GP2** and **Quino-OMe** was obtained as a red powder. Yield: 81.9 mg, 0.11 mmol, 32%. Mp: > 400 °C (decomposition). ^1H NMR (THF-d_8, 300 MHz, 300 K): δ = 7.01-6.94 (m, 8H), 6.77-6.73 (m, 4H), 6.56-6.48 (s, 2H), 6.42-6.34 (m, 2H), 6.23-6.14 (m, 2H), 6.00-5.91 (m, 2H), 3.87-3.85 (s, 2H), 3.85-3.82 (s, 6H), 2.28-2.24 (s, 6H), 1.97-1.92 (s, 12H) ppm. ^{13}C {^1H} NMR (THF-d_8, 101 MHz, 295 K): δ = 160.5, 144.8, 141.9, 137.6, 136.9, 133.7, 133.1, 132.0, 131.4, 128.8, 126.8, 126.2, 123.8, 121.7, 116.7, 114.0, 96.6,

55.8, 21.1, 19.9 ppm. IR: ṽ = 2913, 2852, 1506, 1324, 1236, 830, 738, 548 cm^{-1}. HRMS (MALDI) m/z: $[M]^{+}$: calcd. for $C_{52}H_{46}N_2O_2$: 730.3559; found 730.3572; correct isotope distribution.

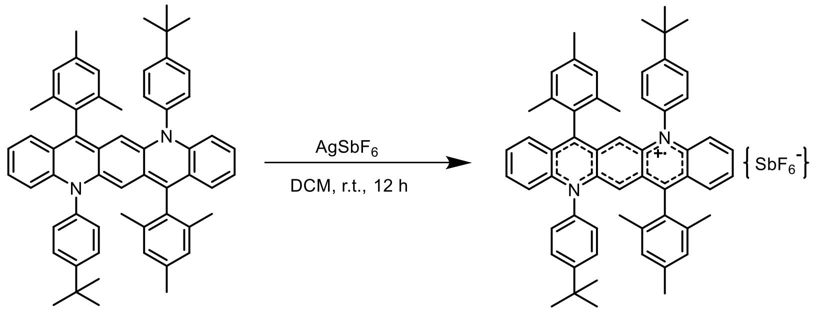

Quino-tBu$^{+\bullet}$ (90%)

Quino-tBu$^{+\bullet}$

To a stirring solution of **Quino-tBu** (50.0 mg, 63.9 µmol, 1.00 eq.) in 10 ml DCM, $AgSbF_6$ (22.0 mg, 63.9 µmol, 1.00 eq.) in 1 ml CH_3CN solution was added dropwise and the reaction mixture was stirred for 12 h at r.t. After that, the mixture was filtered and the solvent was removed under reduced pressure to give radical cation as a dark brown solid. Yield: 58.5 mg, 57.5 µmol, 90%. IR: ṽ = 3057, 2958, 2917, 2867, 1556, 1385, 1248, 1160, 761, 643, 564, 503 cm^{-1}.

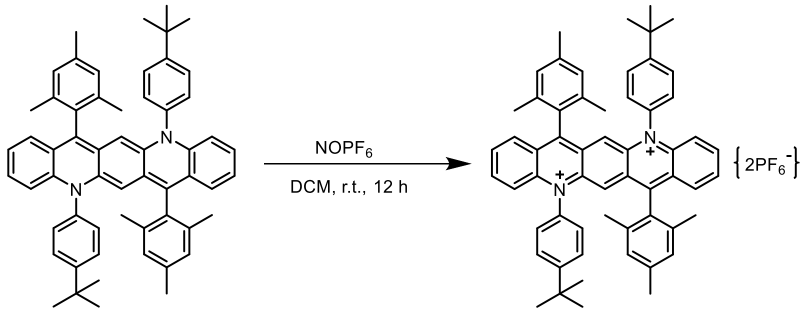

Quino-tBu^{2+} (quant.)

Quino-tBu^{2+}

To a stirring solution of **Quino-tBu** (50.0 mg, 63.9 µmol, 1.00 eq.) in 10 ml DCM, $NOPF_6$ (22.3 mg, 128 µmol, 2.00 eq.) in 1 ml CH_3CN solution was added dropwise and the reaction mixture was stirred for 12 h at r.t.. After that, the mixture was filtered and the solvent was removed under reduced pressure to give the dication as a deep green solid. Yield: 68.5 mg, 63.8 µmol, 100%. ^{1}H NMR (acetonitrile-d_3, 600 MHz, 295 K): δ = 8.33-8.28 (m, 2H), 8.26-8.22 (m, 2H), 7.87-7.81 (m, 8H), 7.71-7.68 (m, 2H), 7.60-7.55 (m, 4H), 7.16-7.12 (m, 4H), 2.47-2.39 (s, 6H), 1.78-1.71 (m, 12H), 1.51-1.48 (s, 6H) ppm. ^{13}C {^{1}H} NMR (acetonitrile-d_3, 151 MHz, 295 K): δ = 167.1, 157.1, 147.5, 144.5, 141.9, 138.0, 137.3, 134.9, 130.8, 130.4, 129.8, 129.3, 129.2, 129.1, 128.2, 123.4, 121.5, 36.0, 31.6, 21.8, 20.3 ppm. IR: ṽ = 2958, 2920, 2867, 1609, 1533, 826, 765, 560 cm^{-1}.

S2. Electron Spin Resonance Spectra

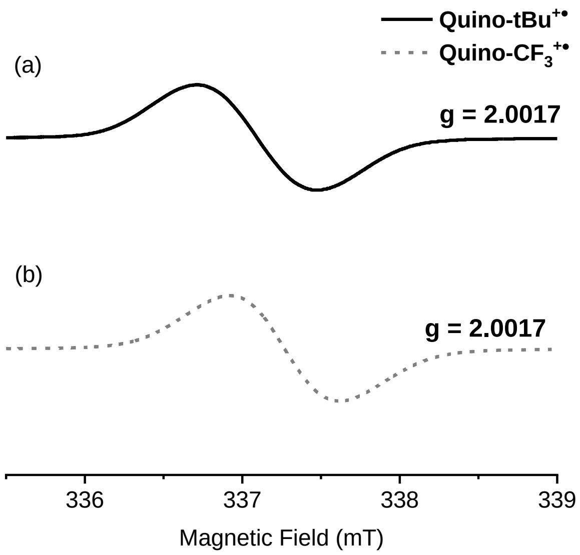

Figure S1. Electron spin resonance (ESR) spectra of a) **Quino-tBu**$^{+\cdot}$ and b) **Quino-CF$_3$**$^{+\cdot}$ recorded in DCM at r.t..

S3. Absorption and Emission Spectra

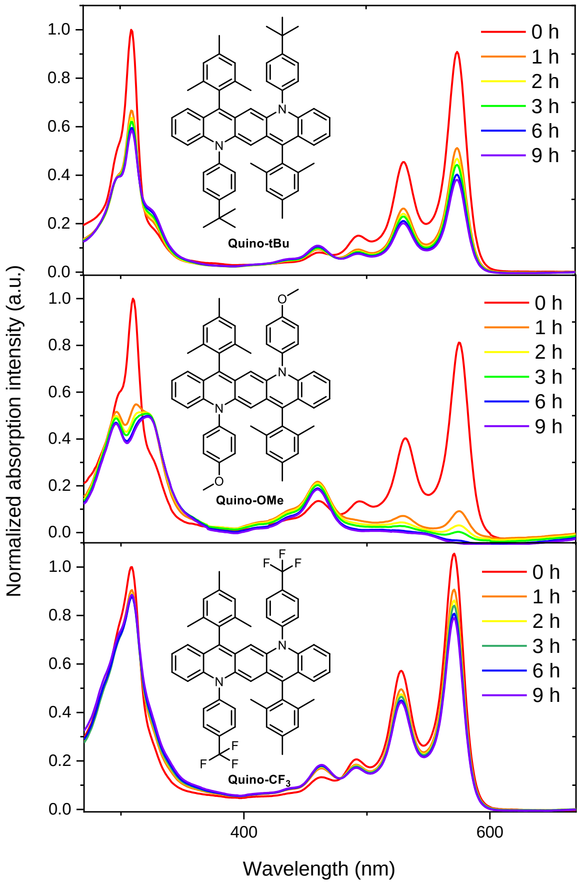

Figure S2. Time-dependent absorption spectra of **Quino-tBu**, **Quino-OMe**, and **Quino-CF$_3$** measured in DCM under ambient conditions.

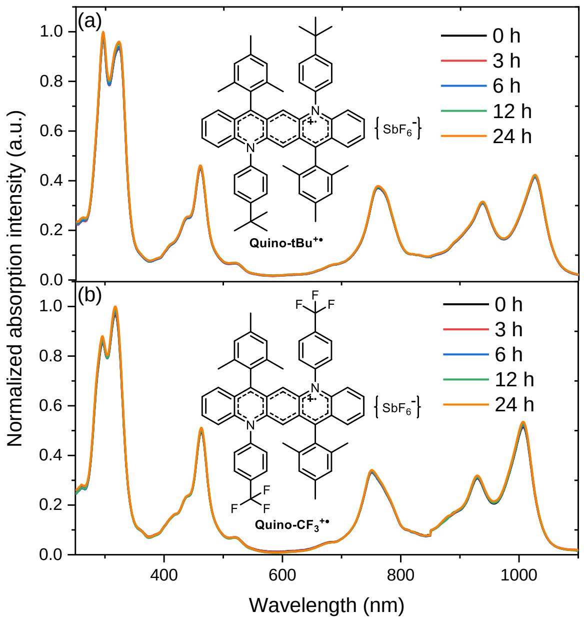

Figure S3. Time-dependent absorption spectra of a) **Quino-tBu$^{+•}$** and b) **Quino-CF$_3^{+•}$** measured in DCM under ambient conditions.

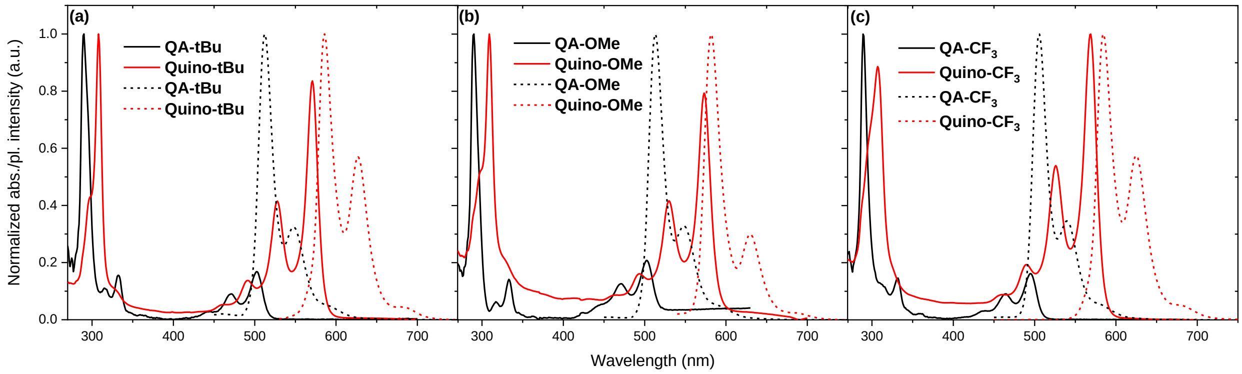

Figure S4. Normalized absorption (solid) and photoluminescence (dashed) spectra of a) **QA-tBu** and **Quino-tBu**; b) **QA-OMe** and **Quino-OMe**; c) **QA-CF$_3$** and **Quino-CF$_3$** measured in THF.

S4. Theoretical Studies

Table S1. (TD)-DFT results at B3LYP/6-311G** level using a PCM model for DCM solvation for neutral, cationic and dicationic **Quino-CF$_3$**.

**Molecules**	**Quino-CF$_3$**				**Quino-CF$_3^+$**				**Quino-CF$_3^{2+}$**			
**Ground State**												
E	-2714.9418664				-2714.7860648				-2714.5917678			
IP	**0**				4.2396				9.5267			
E_{HOMO}	-4.353				-5.393 (α), -6.414 (β)				-7.437			
E_{LUMO}	-2.007				-3.145 (α), -4.124 (β)				-5.179			
E_{H-L}	2.346				2.247 (α), 2.597 (β)				2.258			
**Excited State**												
**Bright state**	**State**	**eV**	**nm**	**f**	**State**	**eV**	**nm**	**f**	**State**	**eV**	**nm**	**f**
**BS1**	**S1**	2.22	559	0.9445	**D1**	1.48	840	0.1263	**S5**	1.90	653	0.1055
**Contribution**	HOMO -> LUMO, 97%				HOMO-β -> LUMO-β, 79%				HOMO-4 -> LUMO, 79%			
**BS2**					**D2**	1.63	761	0.2554	**S12**	3.07	404	0.6858
**Contribution**					HOMO-α -> LUMO-α, 78%				HOMO-8 -> LUMO, 92%			
**BS3**					**D10**	2.83	437	0.3435				
**Contribution**					HOMO-β-6 -> LUMO-β, 78%							

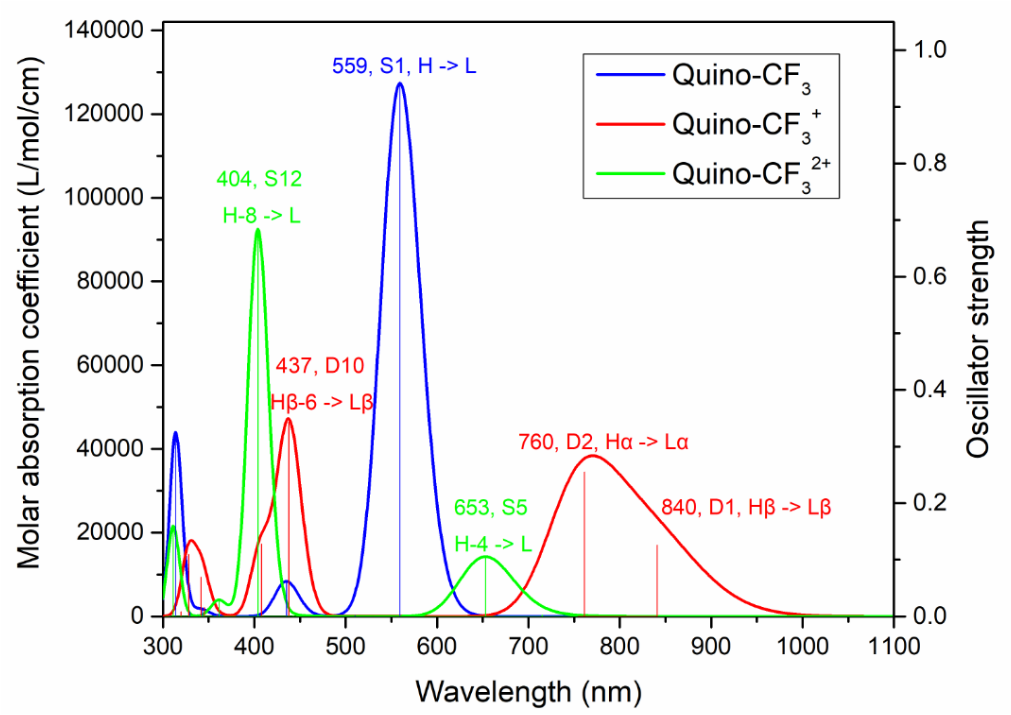

Figure S5. Simulated absorption spectra of neutral, cationic and dicationic **Quino-CF_3** computed at TD-DFT/B3LYP/6-311G** level. All spectra were broadened using Gaussian functions with a full-width at half maximum of 0.2 eV.

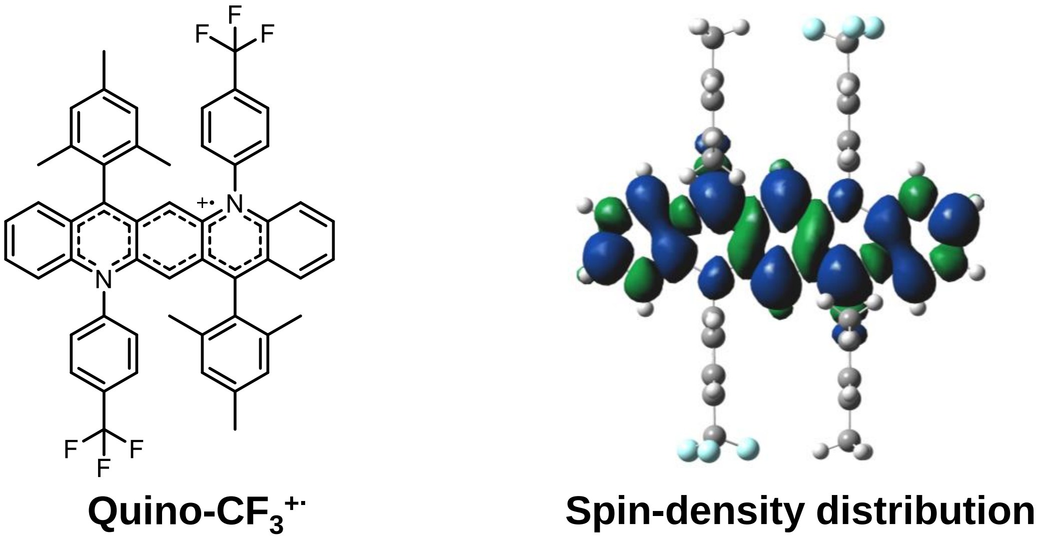

Figure S6. Molecular structure and spin-density distribution of **Quino-$CF_3^{+\cdot}$** calculated at DFT/B3LYP/6-311G** level.

Table S2. Comparison of most significant single excitation contributions of the bright states (BS) for **Quino-CF$_3$** versus **TAP^{2-}**, **Quino-CF$_3$$^{1+}$** versus **TAP^{1-}**, and **Quino-CF$_3$$^{2+}$** versus **TAP**. The MO numbers are indicated below the MO pictures, where the MO numbers in respective neutral species are written in parentheses. The **Quino-CF$_3$** and **TAP** species were computed at B3LYP/6-311G**/DCM and CAM-B3LYP/6-311++G**/THF level of theory, respectively.

State	Most Significant Single Excitation Contribution					
	Quino-CF$_3$			TAP^{2-}		
**BS1**	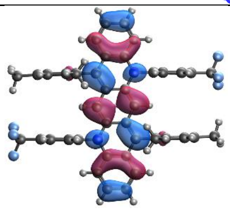<br>**HOMO**<br>(HOMO)	→	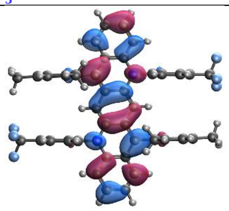<br>**LUMO**<br>(LUMO)	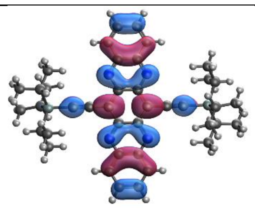<br>**HOMO**<br>(LUMO)	→	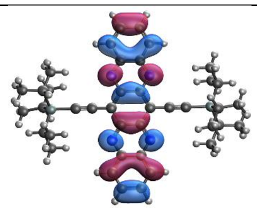<br>**LUMO**<br>(LUMO+1)
	Quino-CF$_3$$^{+\cdot}$			TAP^{1-}		
**BS1**	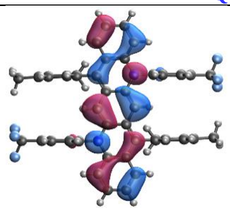<br>**HOMOβ**<br>(HOMO-1)	→	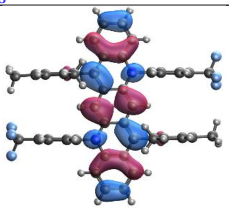<br>**LUMOβ**<br>(HOMO)	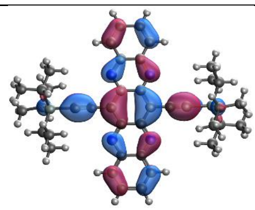<br>**HOMOβ**<br>(HOMO)	→	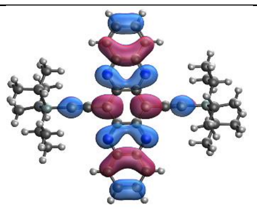<br>**LUMOβ**<br>(LUMO)
**BS2**	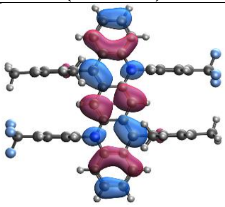<br>**HOMOα**<br>(HOMO)	→	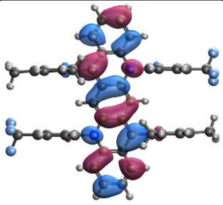<br>**LUMOα**<br>(LUMO)	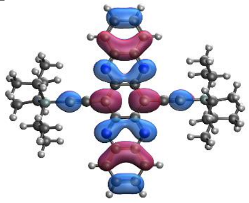<br>**HOMOα**<br>(LUMO)	→	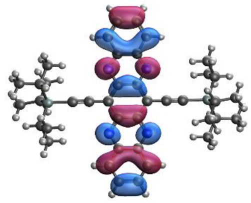<br>**LUMOα**<br>(LUMO+1)
**BS3**	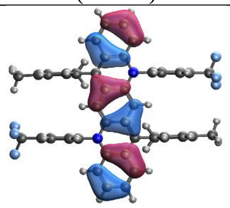<br>**HOMOβ-6**<br>(HOMO-7)	→	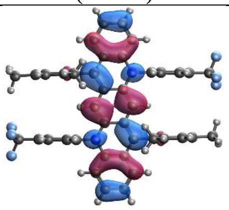<br>**LUMOβ**<br>(HOMO)	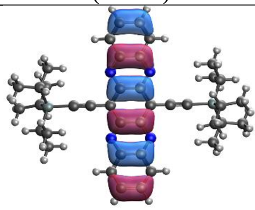<br>**HOMOβ-2**<br>(HOMO-3)	→	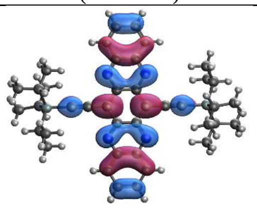<br>**LUMOβ**<br>(LUMO)
	Quino-CF$_3$$^{2+}$			TAP		
**BS1**	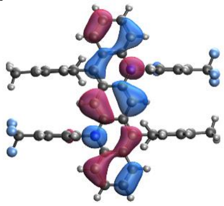<br>**HOMO-4**<br>(HOMO-1)	→	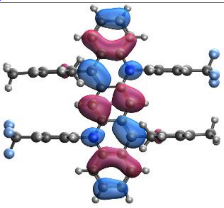<br>**LUMO**<br>(HOMO)	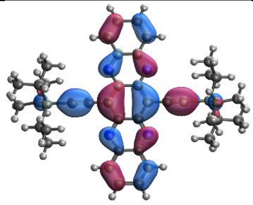<br>**HOMO**<br>(HOMO)	→	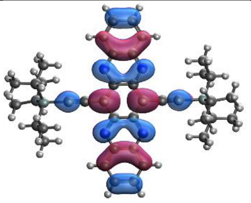<br>**LUMO**<br>(LUMO)
**BS2**	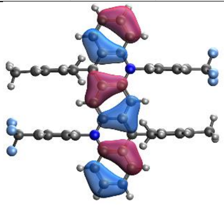<br>**HOMO-8**<br>(HOMO-7)	→	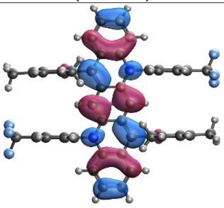<br>**LUMO**<br>(HOMO)	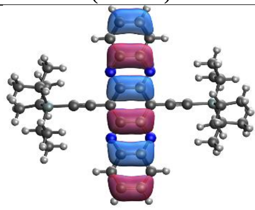<br>**HOMO-3**<br>(HOMO-3)	→	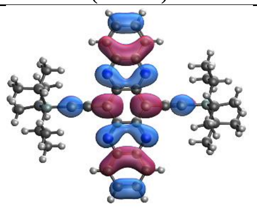<br>**LUMO**<br>(LUMO)

S5. Electrochemistry

The cyclic voltammetry (CV) experiments were carried out using a gold working electrode, a platinum/titanium wire auxiliary electrode, a silver wire reference electrode, a 0.1 M NBu_4PF_6 solution in degassed DCM, and ferrocene/ferrocenium as the reference redox system and internal standard (−4.8 eV).[1]

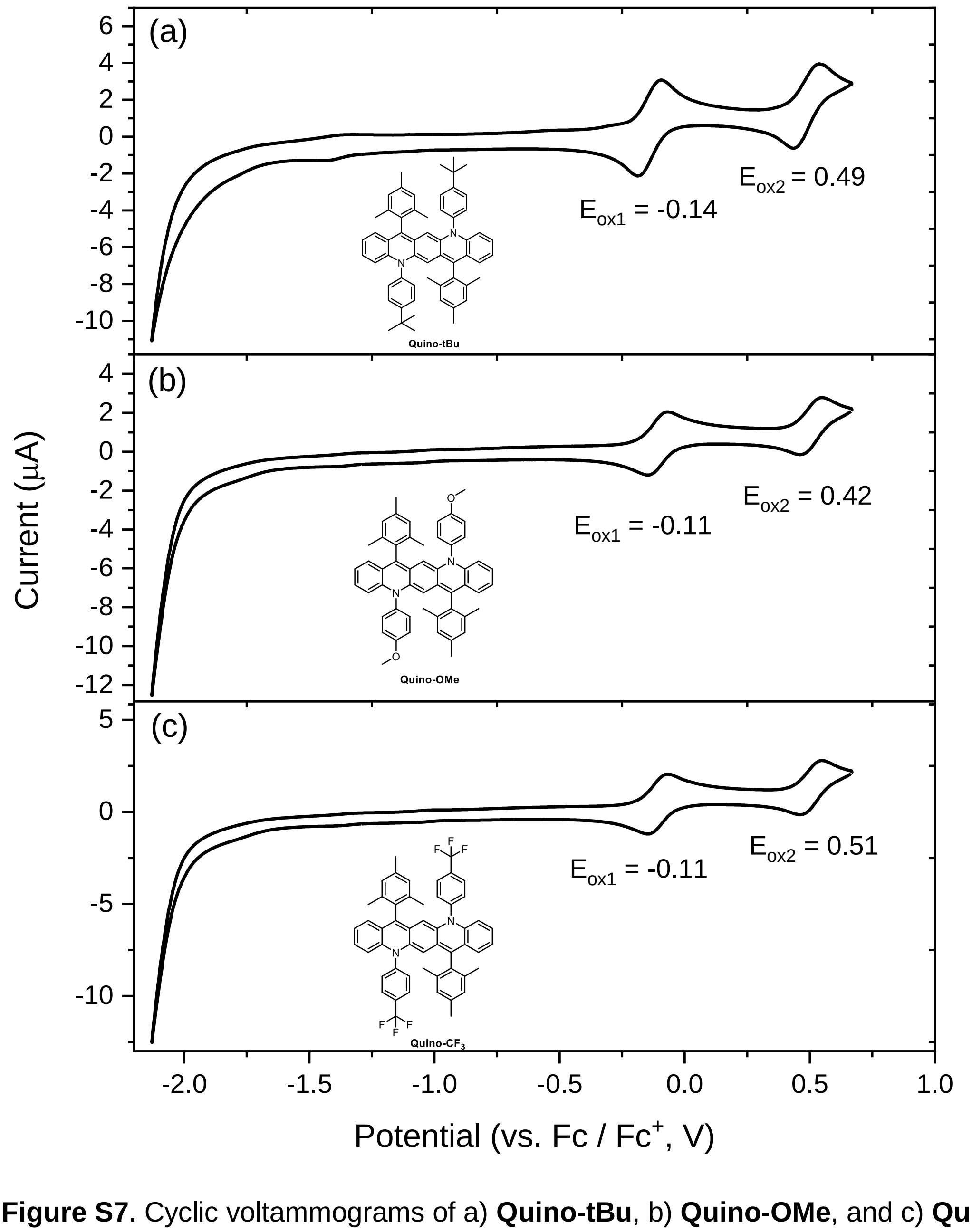

Figure S7. Cyclic voltammograms of a) **Quino-tBu**, b) **Quino-OMe**, and c) **Quino-CF$_3$**.

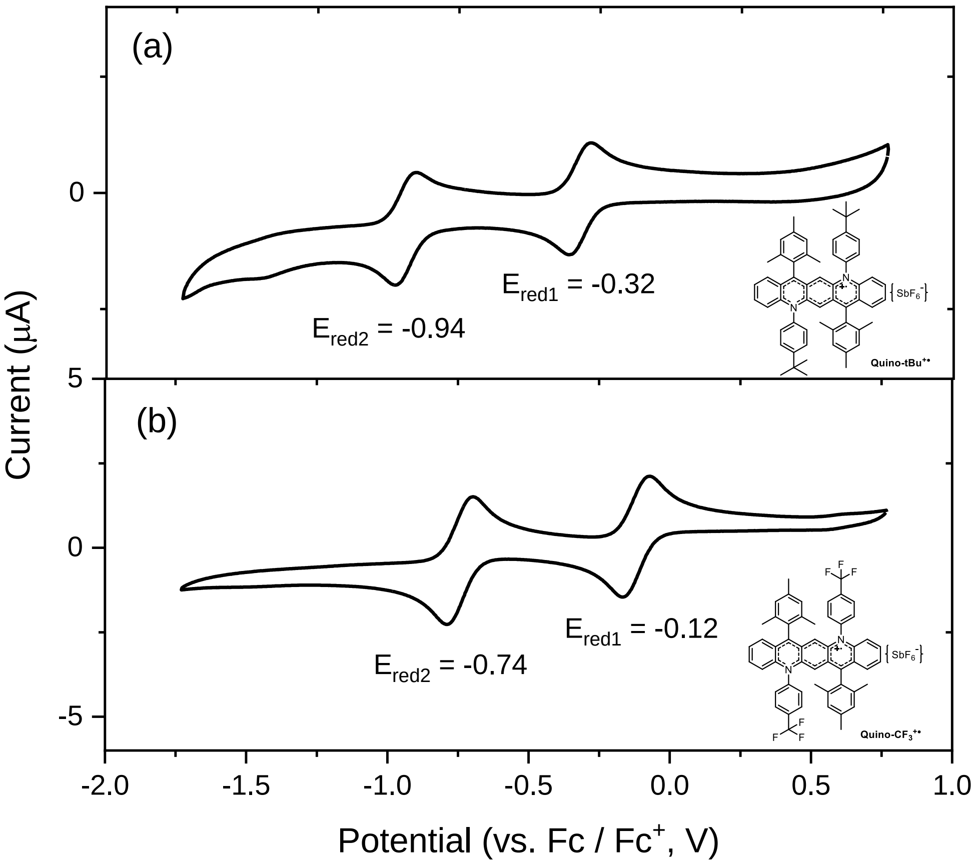

Figure S8. Cyclic voltammograms of a) **Quino-tBu$^{+\cdot}$** and b) **Quino-CF$_3^{+\cdot}$**.

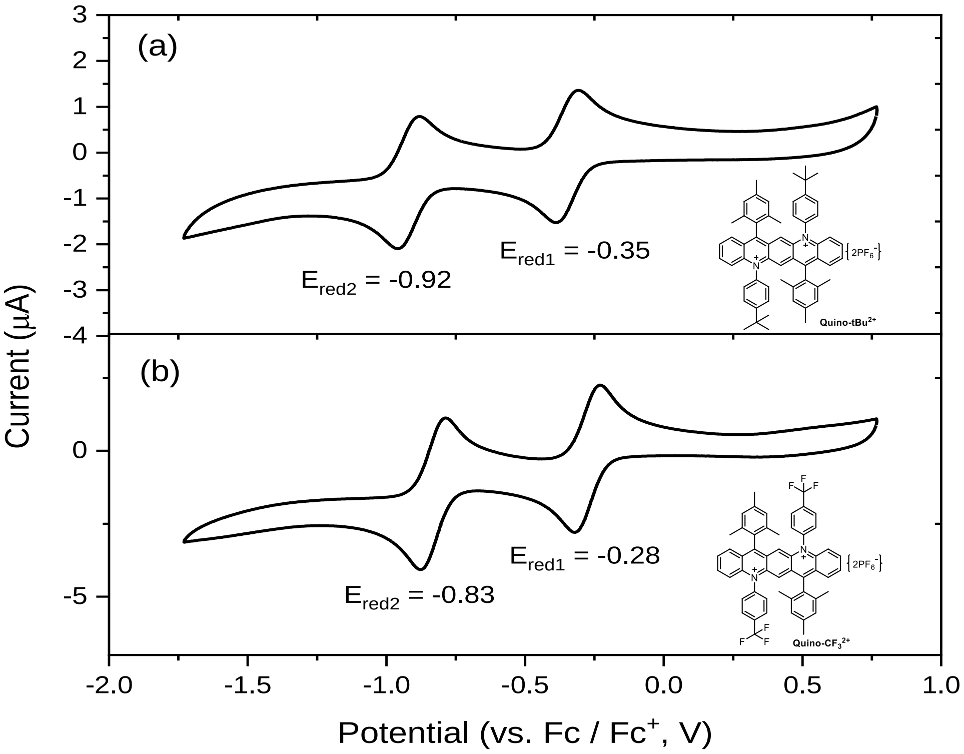

Figure S9. Cyclic voltammograms of a) **Quino-tBu^{2+}** and b) **Quino-CF$_3^{2+}$**.

S6. NMR Spectroscopy

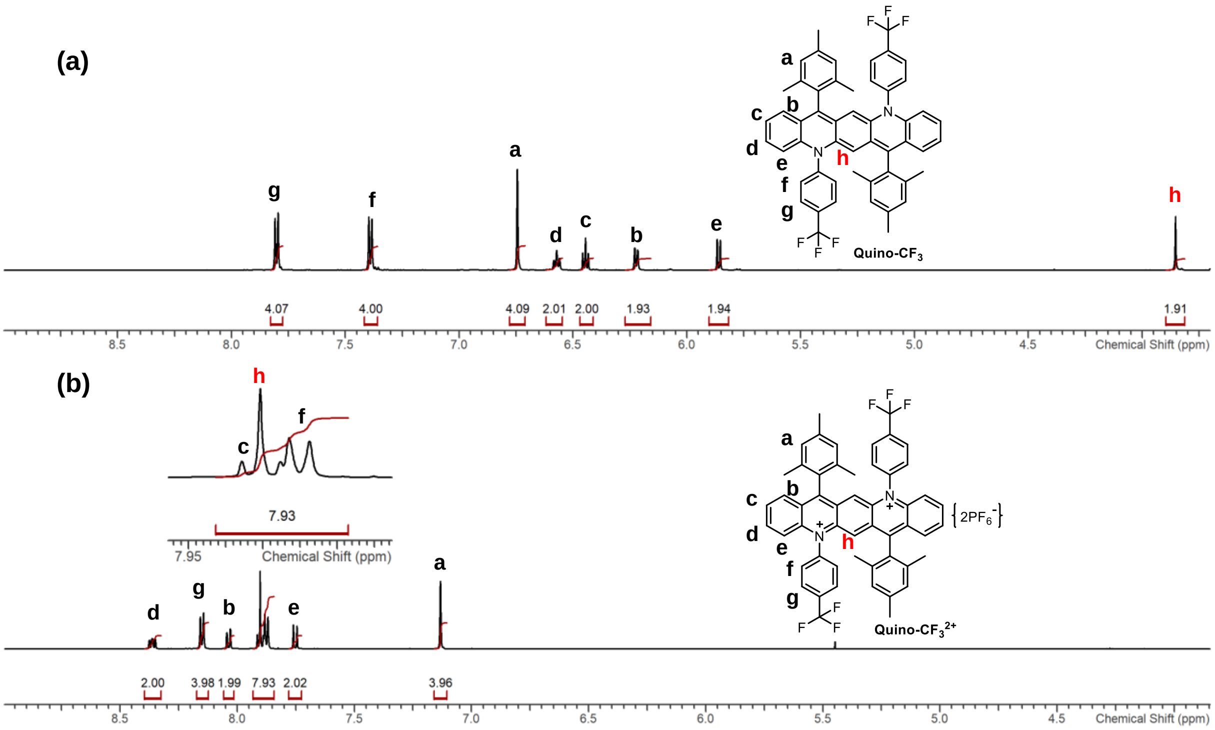

Figure S10. H[1] NMR spectra of a) **Quino-CF_3** and b) **Quino-CF_3^{2+}** in aromatic region.

NMR Spectra

N,N'-Bis(4-*tert*-butylphenyl)quinacridone (**QA-tBu**)

^{1}H NMR

Multiplets Integrals Sum	37.05	Number of Nuclei	66 H's

Acquisition Time (sec)	2.7263	Comment	GZ 359	D	0.5	D1	0.5	DE	10
DS	2	Date	27 Mar 2019 23:33:43			Date Stamp	27 Mar 2019 23:33:43		
File Name	C:\Users\bunz\Application Data\SSH\temp\c190327ubgz.359\2\PDATA\1\1r					Frequency (MHz)	400.3300	GB	0
INSTRUM	<spect>	LB	0.3	NS	128	Nucleus	1H	Number of Transients	128
Origin	spect	Original Points Count	32768	Owner	ns	PC	1		
PROBHD	<Z130030_0004 (CPP BBO 400S1 BB-H&F-D-05 LT)>					PULPROG	<zg30>	Points Count	65536
Pulse Sequence	zg30	Receiver Gain	724.00	SF	400.33004886814			SFO1	400.3320009
SI	65536	SSB	0	SW(cyclical) (Hz)	12019.23	SWH	12019.2307692308		
Solvent	DICHLOROMETHANE-d2			Spectrum Offset (Hz)	1985.5005	Spectrum Type	standard	Sweep Width (Hz)	12019.05
TD	65536	TD0	16	TE	294.999	Temperature (degree C)	21.999	UNC1	<1H>
WDW	1								

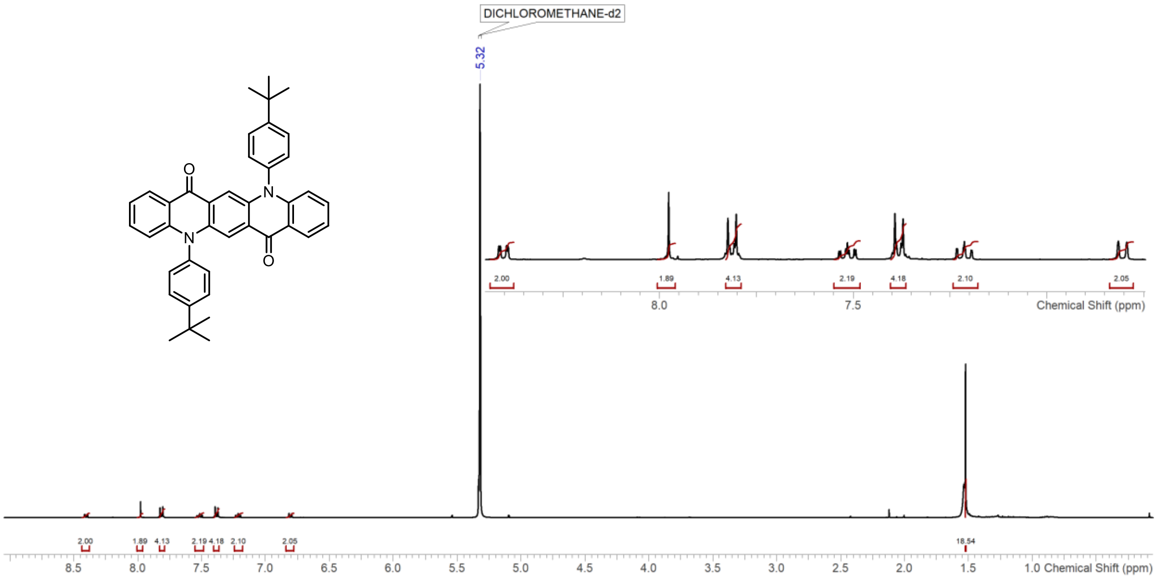

$^{13}C\ \{^{1}H\}$ NMR

Multiplets Integrals Sum	0.00	Number of Nuclei	0 C's

Acquisition Time (sec)	1.5897	Comment	GZ 359	D	1.5	D1	1.5	DE	18
DS	2	Date	27 Mar 2019 23:25:52			Date Stamp	27 Mar 2019 23:25:52		
File Name	\\bunz22\Mitarbeiter\Gaozhan XIE\03_analytics\01-NMR\nmr02\c190327ubgz.359\1\PDATA\1\1r					Frequency (MHz)	100.6631	GB	0
INSTRUM	<spect>	LB	1	NS	4096	Nucleus	13C	Number of Transients	4096
Origin	spect	Original Points Count	49066	Owner	ns	PC	1.4		
PROBHD	<Z130030_0004 (CPP BBO 400S1 BB-H&F-D-05 LT)>			PULPROG	<zgpg30>	Points Count	65536	Pulse Sequence	zgpg30
Receiver Gain	2050.00	SF	100.663059	SFO1	100.67413193649			SI	65536
SSB	0	SW(cyclical) (Hz)	30864.20	SWH	30864.1975308642				
Solvent	DICHLOROMETHANE-d2			Spectrum Offset (Hz)	11135.0059	Spectrum Type	standard	Sweep Width (Hz)	30863.73
TD	98132	TD0	512	TE	295.0014	Temperature (degree C)	22.001	UNC1	<13C>
WDW	1								

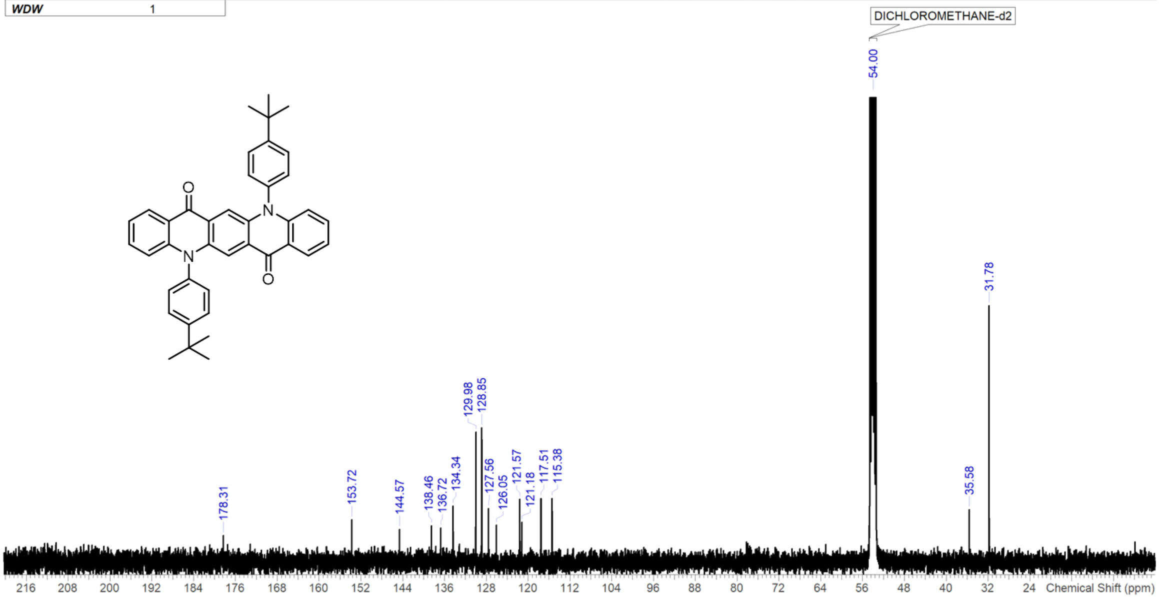

N,N'-Bis(4-methoxylphenyl)quinacridone (**QA-OMe**)

^{1}H NMR

Multiplets Integrals Sum	81.87	*Number of Nuclei*	86 H's

Acquisition Time (sec)	3.6351	*Comment*	GZ 376	*D*	0.1	*D1*	0.1	*DE*	12
DS	2	*Date*	13 Mar 2019 19:19:45			*Date Stamp*	13 Mar 2019 19:19:45		
File Name	\\bunz22\Mitarbeiter\Gaozhan XIE\03_analytics\01-NMR\nmr02\e190313ubgz.376\2\PDATA\1\1r					*Frequency (MHz)*	600.2438	*GB*	0
INSTRUM	<spect>	*LB*	0.3	*NS*	80	*Nucleus*	1H	*Number of Transients*	80
Origin	spect	*Original Points Count*	65536	*Owner*	ns	*PC*	1		
PROBHD	<Z132808_0001 (CP QCI 600S3 H/P/C-N-D-05 Z LT)>					*PULPROG*	<zg30>	*Points Count*	65536
Pulse Sequence	zg30	*Receiver Gain*	15.35	*SF*	600.243829	*SFO1*	600.246830219145		
SI	65536	*SSB*	0	*SW(cyclical) (Hz)*	18028.85	*SWH*	18028.8461538462		
Solvent	DICHLOROMETHANE-d2			*Spectrum Offset (Hz)*	2806.3765	*Spectrum Type*	standard	*Sweep Width (Hz)*	18028.57
TD	131072	*TD0*	10	*TE*	295.0004	*Temperature (degree C)*	22.000	*UNC1*	<1H>
WDW	1								

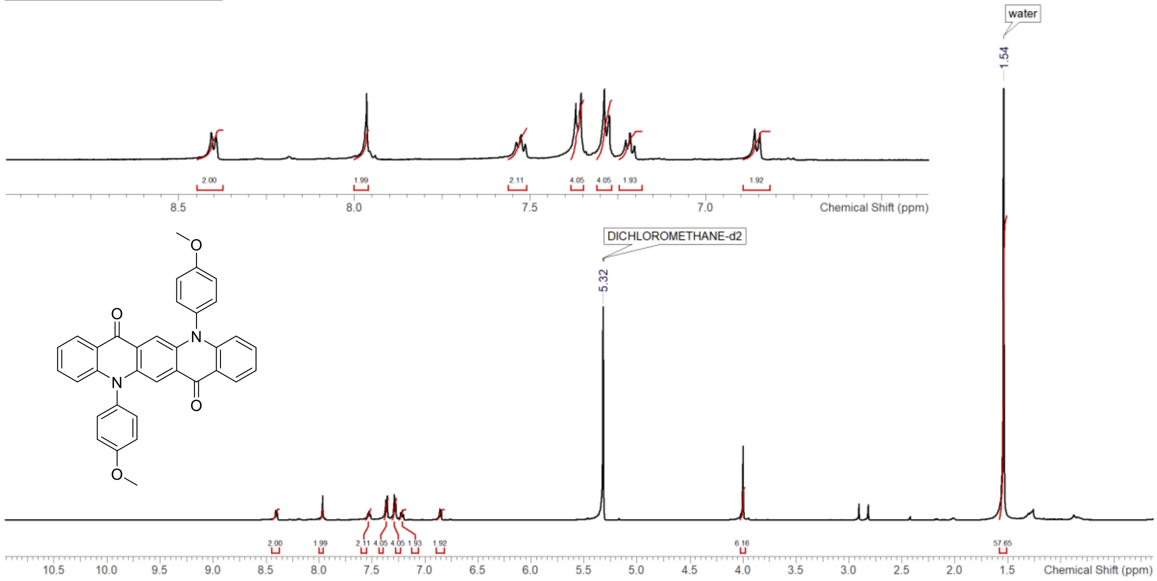

^{13}C {^{1}H} NMR

Multiplets Integrals Sum	0.00	*Number of Nuclei*	0 C's

Acquisition Time (sec)	1.0795	*Comment*	GZ 376	*D*	0.03	*D1*	2	*DE*	18
DS	4	*Date*	13 Mar 2019 19:13:31			*Date Stamp*	13 Mar 2019 19:13:31		
File Name	\\bunz22\Mitarbeiter\Gaozhan XIE\03_analytics\01-NMR\nmr02\e190313ubgz.376\1\PDATA\1\1r					*Frequency (MHz)*	150.9314	*GB*	0
INSTRUM	<spect>	*LB*	1	*NS*	3200	*Nucleus*	13C	*Number of Transients*	3200
Origin	spect	*Original Points Count*	49066	*Owner*	ns	*PC*	1.4		
PROBHD	<Z132808_0001 (CP QCI 600S3 H/P/C-N-D-05 Z LT)>			*PULPROG*	<zgpg30>	*Points Count*	65536	*Pulse Sequence*	zgpg30
Receiver Gain	2050.00	*SF*	150.931431	*SFO1*	150.94803345741			*SI*	65536
SSB	0	*SW(cyclical) (Hz)*	45454.55	*SWH*	45454.5454545455				
Solvent	DICHLOROMETHANE-d2			*Spectrum Offset (Hz)*	16643.5547	*Spectrum Type*	standard	*Sweep Width (Hz)*	45453.85
TD	98132	*TD0*	400	*TE*	294.9997	*Temperature (degree C)*	22.000	*UNC1*	<13C>
WDW	1								

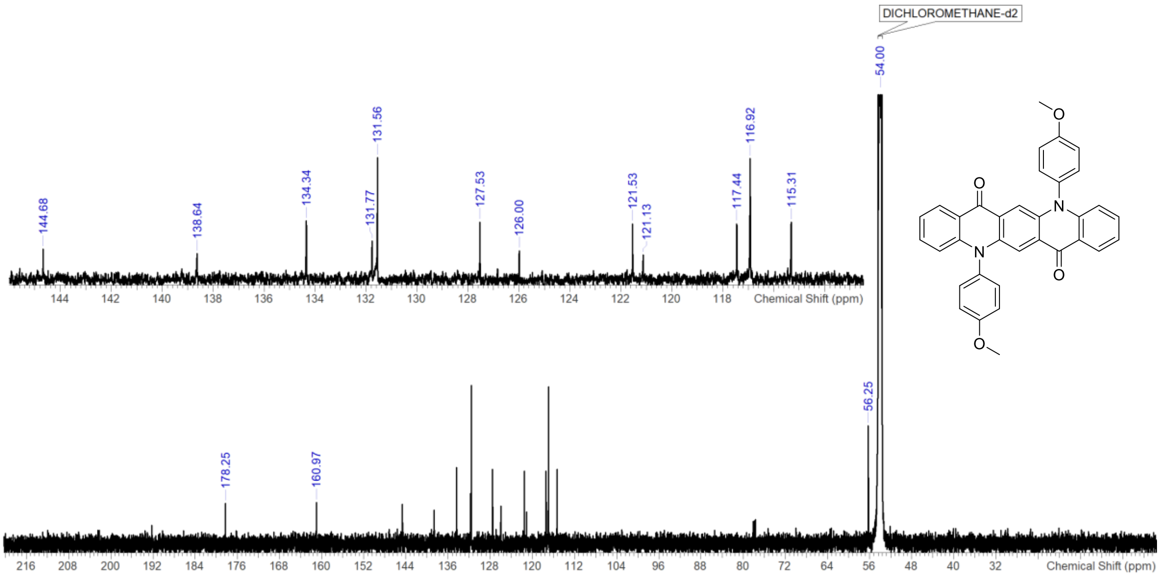

N,N'-Bis(4-trifluoromethylphenyl)quinacridone (**QA-CF_3**)

^{1}H NMR

Multiplets Integrals Sum	17.82	Number of Nuclei	32 H's

Acquisition Time (sec)	2.7263	Comment	GZ 386	D	0.5	D1	0.5	DE	10
DS	2	Date	02 Apr 2019 19:17:32			Date Stamp	02 Apr 2019 19:17:32		
File Name	\\bunz22\Mitarbeiter\Gaozhan XIE\03_analytics\01-NMR\nmr02\c190402ubgz.386\2\PDATA\1\1r					Frequency (MHz)	400.3300	GB	0
INSTRUM	<spect>	LB	0.3	NS	128	Nucleus	1H	Number of Transients	128
Origin	spect	Original Points Count	32768	Owner	ns	PC	1		
PROBHD	<Z130030_0004 (CPP BBO 400S1 BB-H&F-D-05 LT)>					PULPROG	<zg30>	Points Count	65536
Pulse Sequence	zg30	Receiver Gain	645.00	SF	400.33004918049			SFO1	400.3320009
SI	65536	SSB	0	SW(cyclical) (Hz)	12019.23	SWH	12019.2307692308		
Solvent	DICHLOROMETHANE-d2			Spectrum Offset (Hz)	1985.5085	Spectrum Type	standard	Sweep Width (Hz)	12019.05
TD	65536	TD0	16	TE	294.9981	Temperature (degree C)	21.998	UNC1	<1H>
WDW	1								

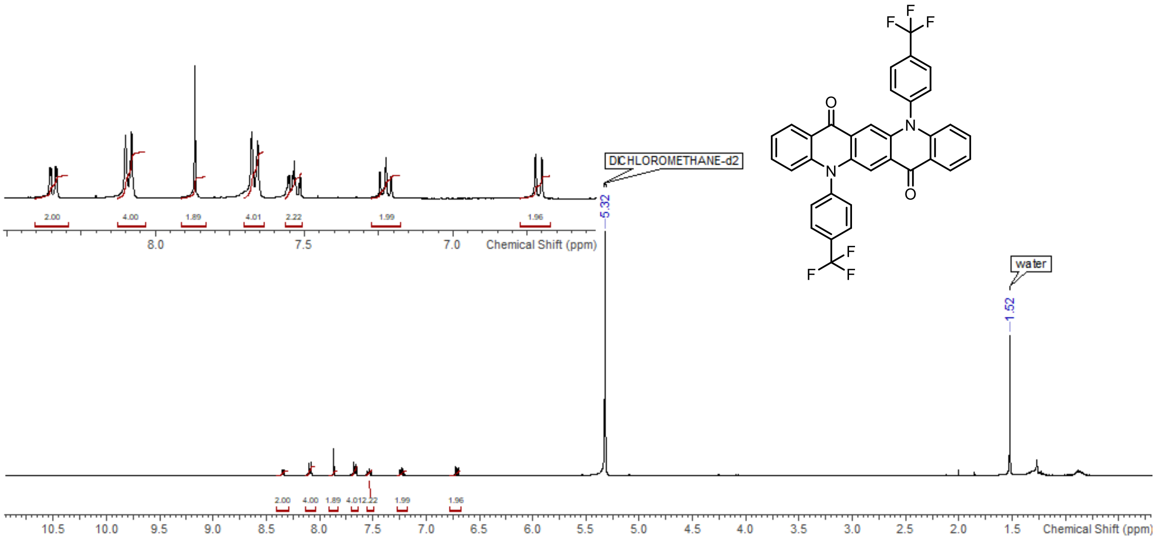

^{13}C {^{1}H} NMR

Multiplets Integrals Sum	0.00	Number of Nuclei	0 C's

Acquisition Time (sec)	1.5897	Comment	GZ 386	D	1.5	D1	1.5	DE	18
DS	2	Date	02 Apr 2019 19:09:41			Date Stamp	02 Apr 2019 19:09:41		
File Name	\\bunz22\Mitarbeiter\Gaozhan XIE\03_analytics\01-NMR\nmr02\c190402ubgz.386\1\PDATA\1\1r					Frequency (MHz)	100.6631	GB	0
INSTRUM	<spect>	LB	1	NS	4096	Nucleus	13C	Number of Transients	4096
Origin	spect	Original Points Count	49066	Owner	ns	PC	1.4		
PROBHD	<Z130030_0004 (CPP BBO 400S1 BB-H&F-D-05 LT)>			PULPROG	<zgpg30>	Points Count	65536	Pulse Sequence	zgpg30
Receiver Gain	1620.00	SF	100.663059	SFO1	100.67413193649			SI	65536
SSB	0	SW(cyclical) (Hz)	30864.20	SWH	30864.1975308642				
Solvent	DICHLOROMETHANE-d2			Spectrum Offset (Hz)	11134.5430	Spectrum Type	standard	Sweep Width (Hz)	30863.73
TD	98132	TD0	512	TE	294.999	Temperature (degree C)	21.999	UNC1	<13C>
WDW	1								

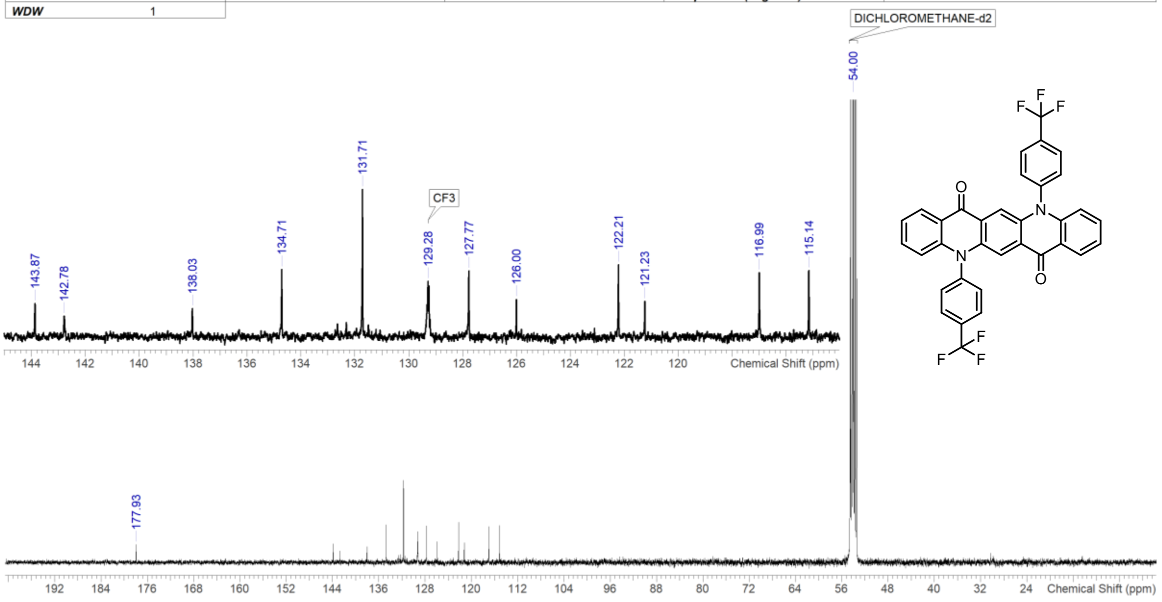

7,14-Dimesityl-5,12-dihydro-5,12-bis(4-tert-butylphenyl)diazapentacene (**Quino-tBu**)

^{1}H NMR

Acquisition Time (sec)	3.6351	***Comment***	GZ 369	***D***	0.1	***D1***	0.1	***DE***	12
DS	2	***Date***	04 Mar 2019 17:11:44			***Date Stamp***	04 Mar 2019 17:11:44		
File Name	\\bunz22\Mitarbeiter\Gaozhan XIE\03_analytics\01-NMR\nmr02\e190304ubgz.369\2\PDATA\1\1r							***Frequency (MHz)***	600.2438
GB	0	***INSTRUM***	<spect>	***LB***	0.3	***NS***	128	***Nucleus***	1H
Number of Transients	128	***Origin***	spect	***Original Points Count***	65536	***Owner***	ns	***PC***	1
PROBHD	<Z132808_0001 (CP QCI 600S3 H/P/C-N-D-05 Z LT)>					***PULPROG***	<zg30>	***Points Count***	65536
Pulse Sequence	zg30	***Receiver Gain***	13.85	***SF***	600.243829	***SFO1***	600.246830219145		
SI	65536	***SSB***	0	***SW(cyclical) (Hz)***	18028.85	***SWH***	18028.8461538462		
Solvent	THF	***Spectrum Offset (Hz)***	2809.0774	***Spectrum Type***	standard	***Sweep Width (Hz)***	18028.57	***TD***	131072
TD0	16	***TE***	295.0003	***Temperature (degree C)***	22.000	***UNC1***	<1H>	***WDW***	1

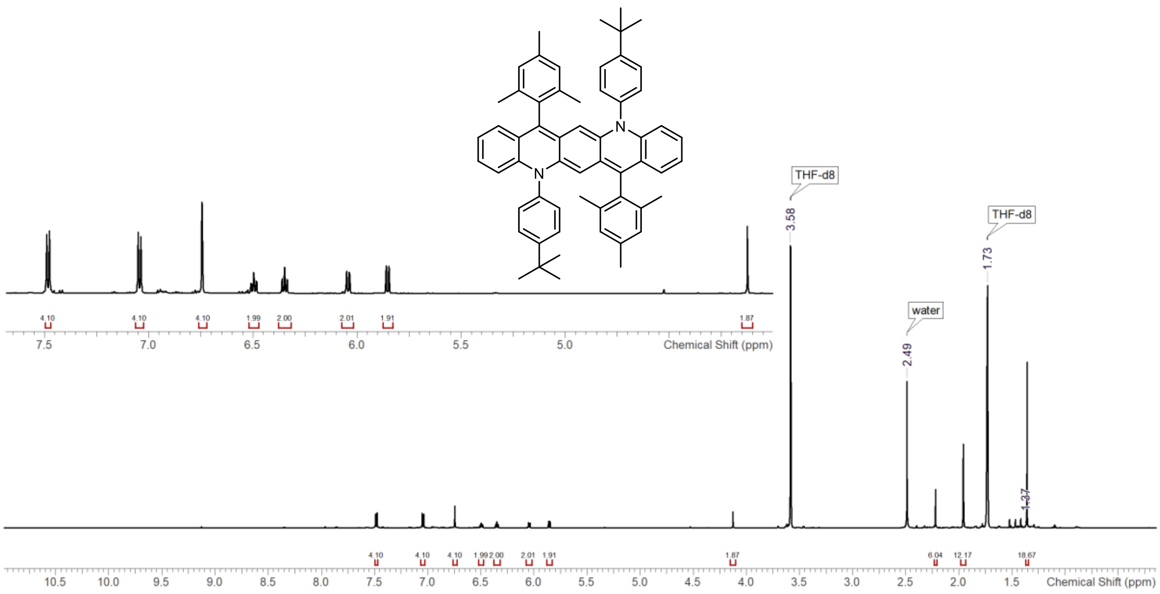

^{13}C {^{1}H} NMR

Multiplets Integrals Sum	0.00	***Number of Nuclei***	0 C's

Acquisition Time (sec)	1.0795	***Comment***	GZ 369	***D***	0.03	***D1***	2	***DE***	18
DS	4	***Date***	04 Mar 2019 17:02:37			***Date Stamp***	04 Mar 2019 17:02:37		
File Name	\\bunz22\Mitarbeiter\Gaozhan XIE\03_analytics\01-NMR\nmr02\e190304ubgz.369\1\PDATA\1\1r					***Frequency (MHz)***	150.9314	***GB***	0
INSTRUM	<spect>	***LB***	1	***NS***	4096	***Nucleus***	13C	***Number of Transients***	4096
Origin	spect	***Original Points Count***	49066	***Owner***	ns	***PC***	1.4		
PROBHD	<Z132808_0001 (CP QCI 600S3 H/P/C-N-D-05 Z LT)>					***PULPROG***	<zgpg30>	***Points Count***	65536
Pulse Sequence	zgpg30	***Receiver Gain***	2050.00	***SF***	150.931431	***SFO1***	150.94803345741		
SI	65536	***SSB***	0	***SW(cyclical) (Hz)***	45454.55	***SWH***	45454.5454545455		
Solvent	THF	***Spectrum Offset (Hz)***	16678.6387	***Spectrum Type***	standard	***Sweep Width (Hz)***	45453.85	***TD***	98132
TD0	512	***TE***	294.9989	***Temperature (degree C)***	21.999	***UNC1***	<13C>	***WDW***	1

User Notes	GZ 369

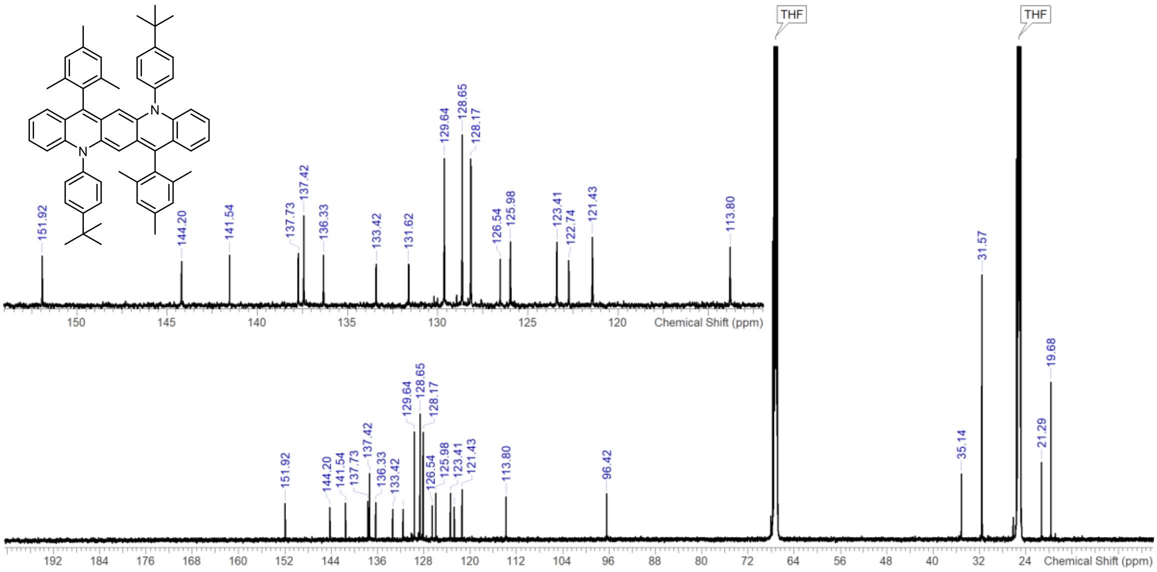

7,14-Dimesityl-5,12-dihydro-5,12-bis(4-methoxylphenyl)diazapentacene (**Quino-OMe**)

^{1}H NMR

Multiplets Integrals Sum	45.71	Number of Nuclei	47 H's

Acquisition Time (sec)	3.6351	*Comment*	Z104275_0201 (PA BBO 300S1 BBF-H-D-05 Z)			*D*	0.1	*D1*	0.1
DE	6.5	*DS*	2	*Date*	15 May 2019 01:03:45			*Date Stamp*	15 May 2019 01:03:45
File Name	\\bunz22\Mitarbeiter\Gaozhan XIE\03_analytics\01-NMR\nmr02\a190514ubxgz378\1\PDATA\1\1r					*Frequency (MHz)*	300.5100	*GB*	0
INSTRUM	<spect>	*LB*	0.3	*NS*	128	*Nucleus*	1H	*Number of Transients*	128
Origin	spect	*Original Points Count*	32768	*Owner*	ns	*PC*	1		
PROBHD	<Z104275_0201 (PA BBO 300S1 BBF-H-D-05 Z)>			*PULPROG*	<zg30>	*Points Count*	65536	*Pulse Sequence*	zg30
Receiver Gain	724.00	*SF*	300.510043559377			*SFO1*	300.51150255		
SI	65536	*SSB*	0	*SW(cyclical) (Hz)*	9014.42	*SWH*	9014.42307692308		
Solvent	THF	*Spectrum Offset (Hz)*	1492.0177	*Spectrum Type*	standard	*Sweep Width (Hz)*	9014.29	*TD*	65536
TD0	16	*TE*	300.0031	*Temperature (degree C)*	27.003	*UNC1*	<1H>	*WDW*	1

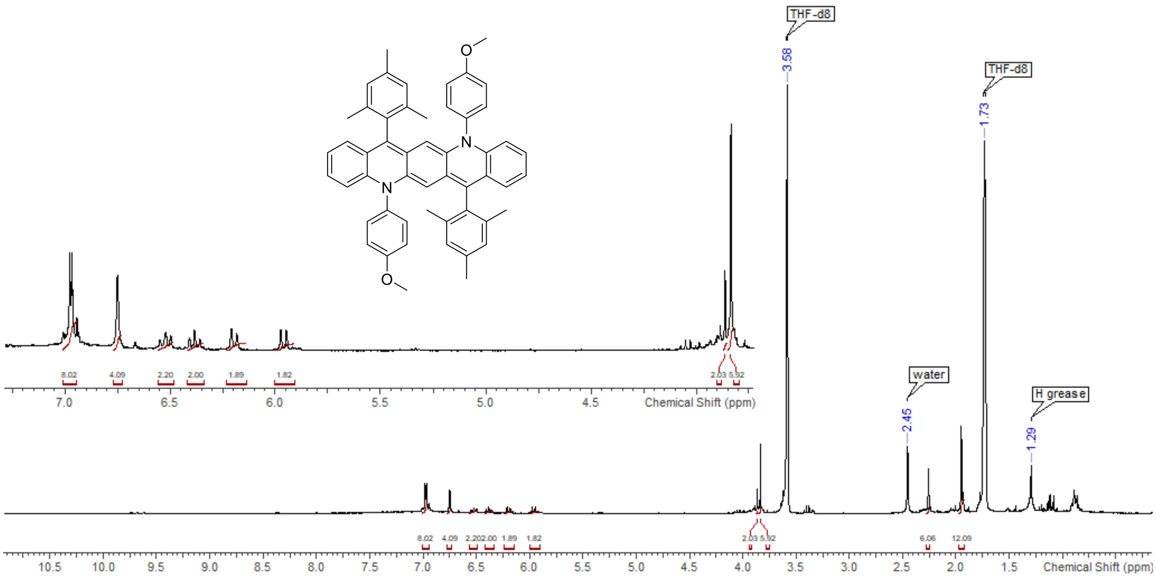

^{13}C $\{^{1}H\}$ NMR

Multiplets Integrals Sum	0.00	Number of Nuclei	0 C's

**Acquisition Time (sec)**	1.5897	**Comment**	GZ 378	**D**	1.5	**D1**	1.5	**DE**	18
**DS**	2	**Date**	29 Mar 2019 05:20:49			**Date Stamp**	29 Mar 2019 05:20:49		
**File Name**	\\bunz22\Mitarbeiter\Gaozhan XIE\03_analytics\01-NMR\nmr02\c190328ubgz.378\1\PDATA\1\1r					**Frequency (MHz)**	100.6631	**GB**	0
**INSTRUM**	<spect>	**LB**	1	**NS**	4096	**Nucleus**	13C	**Number of Transients**	4096
**Origin**	spect	**Original Points Count**	49066	**Owner**	ns	**PC**	1.4		
**PROBHD**	<Z130030_0004 (CPP BBO 400S1 BB-H&F-D-05 LT)>					**PULPROG**	<zgpg30>	**Points Count**	65536
**Pulse Sequence**	zgpg30	**Receiver Gain**	2050.00	**SF**	100.663059	**SFO1**	100.67413193649		
**SI**	65536	**SSB**	0	**SW(cyclical) (Hz)**	30864.20	**SWH**	30864.1975308642		
**Solvent**	THF	**Spectrum Offset (Hz)**	11177.6270	**Spectrum Type**	standard	**Sweep Width (Hz)**	30863.73	**TD**	98132
**TD0**	512	**TE**	295.0005	**Temperature (degree C)**	22.000	**UNC1**	<13C>	**WDW**	1

User Notes	GZ 378

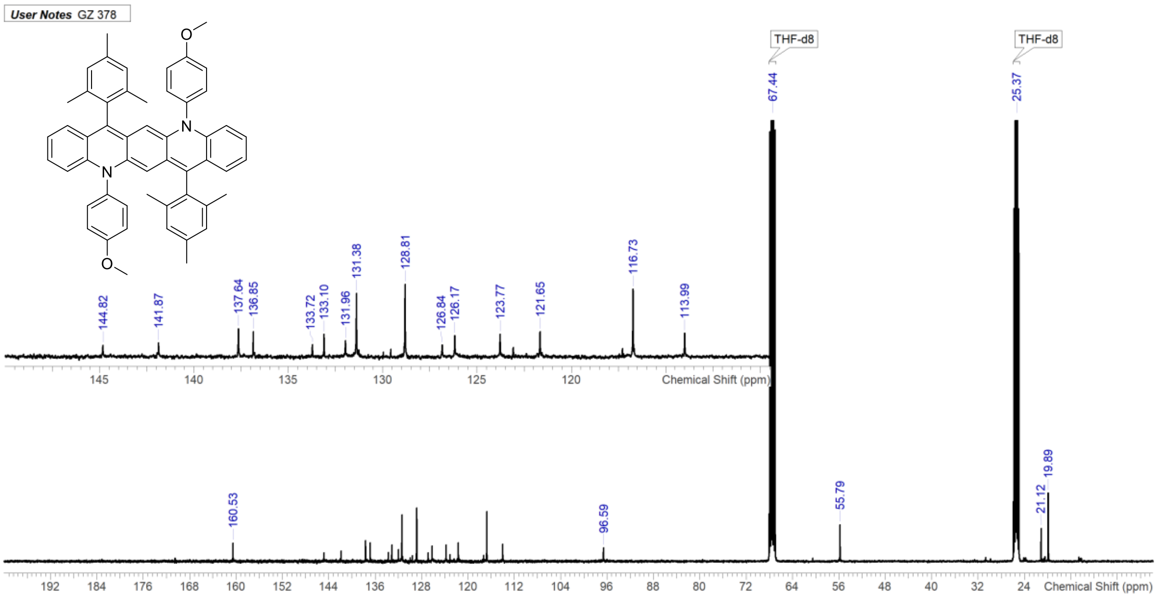

7,14-Dimesityl-5,12-dihydro-5,12-bis(4-trifluoromethylphenyl)diazapentacene (**Quino-CF_3**)

^{1}H NMR

Multiplets Integrals Sum	39.87	Number of Nuclei	40 H's

Acquisition Time (sec)	3.6351	Comment	GZ 388	D	0.1	D1	0.1	DE	12
DS	2	Date	07 Apr 2019 17:45:36			Date Stamp	07 Apr 2019 17:45:36		
File Name	\\bunz22\Mitarbeiter\Gaozhan XIE\03_analytics\01-NMR\nmr02\e190405ubgz.388\2\PDATA\1\1r					Frequency (MHz)	600.2440	GB	0
INSTRUM	<spect>	LB	0.3	NS	128	Nucleus	1H	Number of Transients	128
Origin	spect	Original Points Count	65536	Owner	ns	PC	1		
PROBHD	<Z132808_0001 (CP QCI 600S3 H/P/C-N-D-05 Z LT)>					PULPROG	<zg30>	Points Count	65536
Pulse Sequence	zg30	Receiver Gain	15.35	SF	600.243944334864			SFO1	600.246830219145
SI	65536	SSB	0	SW(cyclical) (Hz)	18028.85	SWH	18028.8461538462		
Solvent	THF	Spectrum Offset (Hz)	3924.3643	Spectrum Type	standard	Sweep Width (Hz)	18028.57	TD	131072
TD0	16	TE	295.0005	Temperature (degree C)	22.000	UNC1	<1H>	WDW	1

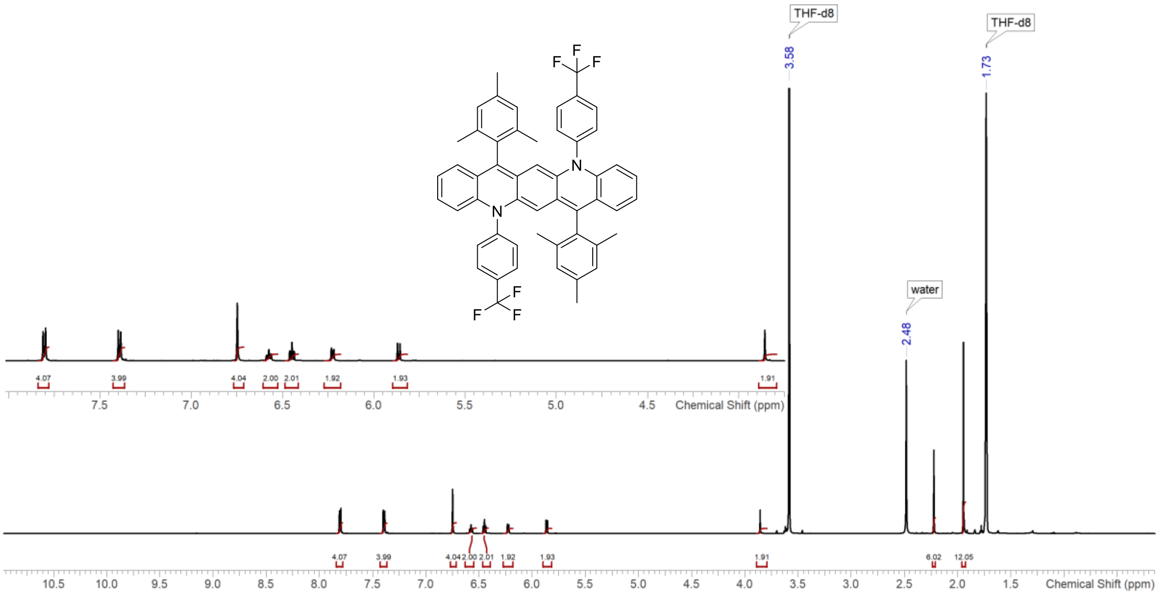

^{13}C NMR

Multiplets Integrals Sum	0.00	Number of Nuclei	0 C's

Acquisition Time (sec)	1.0795	Comment	GZ 388	D	0.03	D1	2	DE	18
DS	4	Date	07 Apr 2019 17:36:36			Date Stamp	07 Apr 2019 17:36:36		
File Name	\\bunz22\Mitarbeiter\Gaozhan XIE\03_analytics\01-NMR\nmr02\e190405ubgz.388\1\PDATA\1\1r					Frequency (MHz)	150.9314	GB	0
INSTRUM	<spect>	LB	1	NS	4096	Nucleus	13C	Number of Transients	4096
Origin	spect	Original Points Count	49066	Owner	ns	PC	1.4		
PROBHD	<Z132808_0001 (CP QCI 600S3 H/P/C-N-D-05 Z LT)>					PULPROG	<zgpg30>	Points Count	65536
Pulse Sequence	zgpg30	Receiver Gain	2050.00	SF	150.931431	SFO1	150.94803345741		
SI	65536	SSB	0	SW(cyclical) (Hz)	45454.55	SWH	45454.5454545455		
Solvent	THF	Spectrum Offset (Hz)	16995.7363	Spectrum Type	standard	Sweep Width (Hz)	45453.85	TD	98132
TD0	512	TE	295.0006	Temperature (degree C)	22.001	UNC1	<13C>	WDW	1

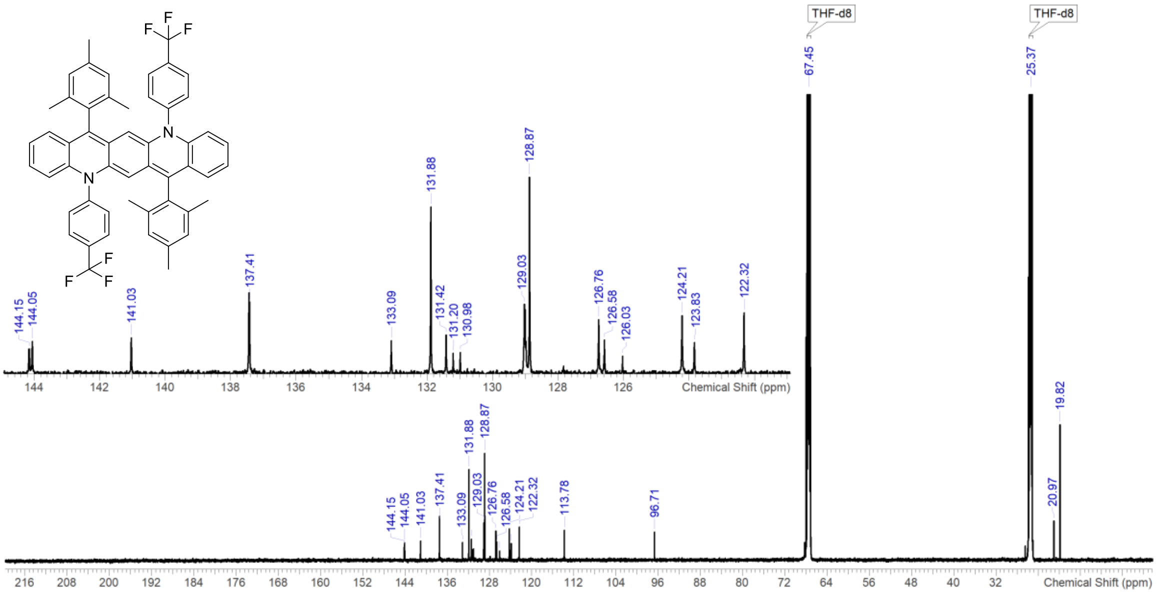

^{1}H, ^{1}H COSY

Acquisition Time (sec)	(0.2908, 0.0364)	Comment	Graf COSYGPSW THF /opt/topspin oci 42
Date	21 May 2019 15:16:00		
File Name	\\bunz22\Mitarbeiter\Gaozhan XIE\03_analytics\01-NMR\nmr02\c190528ubgz.388\40019\PDATA\1\2rr		
Frequency (MHz)	(399.8900, 399.8900)	Nucleus	(1H, 1H)
Number of Transients	1	Origin	spect
Original Points Count	(1024, 128)	Owner	auto
Points Count	(1024, 1024)	Pulse Sequence	cosygpppqf
Solvent	THF	Spectrum Type	COSY
Sweep Width (Hz)	(3517.69, 3517.69)	Temperature (degree C)	22.148
Title	Graf COSYGPSW THF /opt/topspin oci 42		

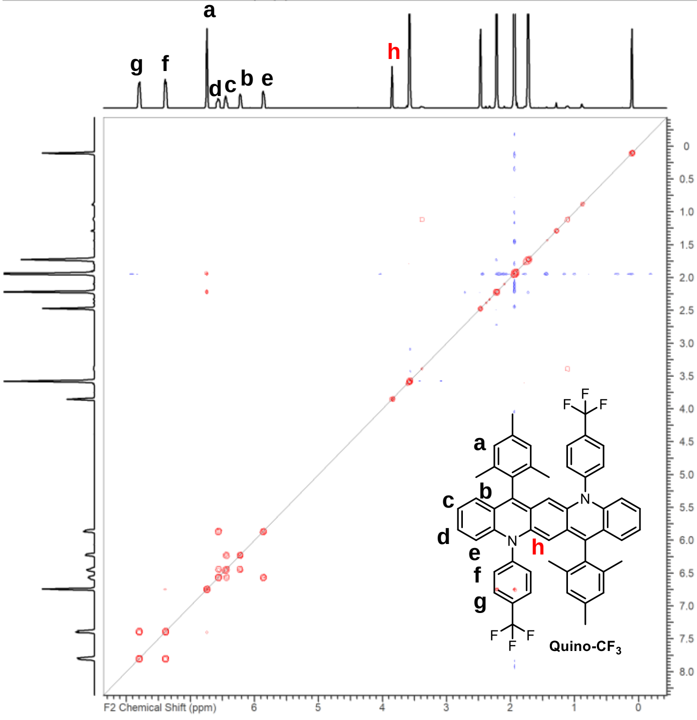

^{1}H, ^{1}H ROESY

Acquisition Time (sec)	(0.2556, 0.0319)	Comment	GZ 388	Date	20 May 2019 01:23:14
File Name	\\bunz22\Mitarbeiter\Gaozhan XIE\03_analytics\01-NMR\nmr02\c190516ubgz.388\3\PDATA\1\2rr				
Frequency (MHz)	(400.1800, 400.1800)			Nucleus	(1H, 1H)
Number of Transients	16	Origin	spect	Original Points Count	(2048, 256)
Owner	ns	Points Count	(2048, 2048)	Pulse Sequence	roesyetgp.2
Solvent	THF	Spectrum Type	ROESY	Sweep Width (Hz)	(8008.91, 8008.91)
Temperature (degree C)	21.998	Title	GZ 388		

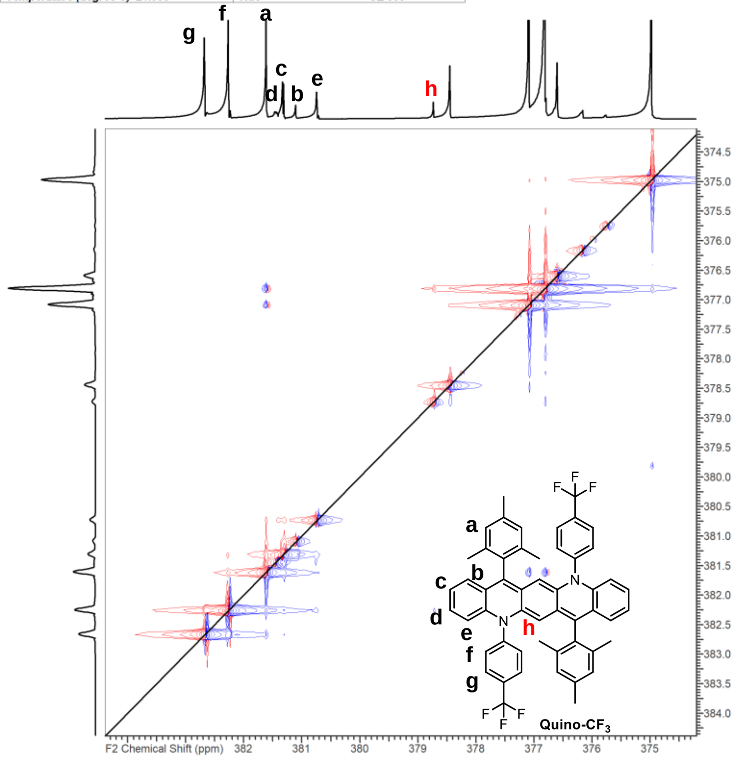

^{1}H, ^{19}F NOESY

Acquisition Time (sec)	(0.2561, 0.0021)	*Comment*	Graf A_19F_HOESY THF /opt/topspin oci 42
Date	21 May 2019 15:45:06		
File Name	\\bunz22\Mitarbeiter\Gaozhan XIE\03_analytics\01-NMR\nmr02\c190528ubgz.388\40021\PDATA\1\2rr		
Frequency (MHz)	(399.8900, 376.2726)	*Nucleus*	(1H, 19F)
Number of Transients	8	*Origin*	spect
Original Points Count	(1024, 64)	*Owner*	auto
Points Count	(1024, 512)	*Pulse Sequence*	hoesyph
Solvent	THF	*Spectrum Type*	NOESY
Sweep Width (Hz)	(3993.96, 30061.65)	*Temperature (degree C)*	22.148
Title	Graf A_19F_HOESY THF /opt/topspin oci 42		

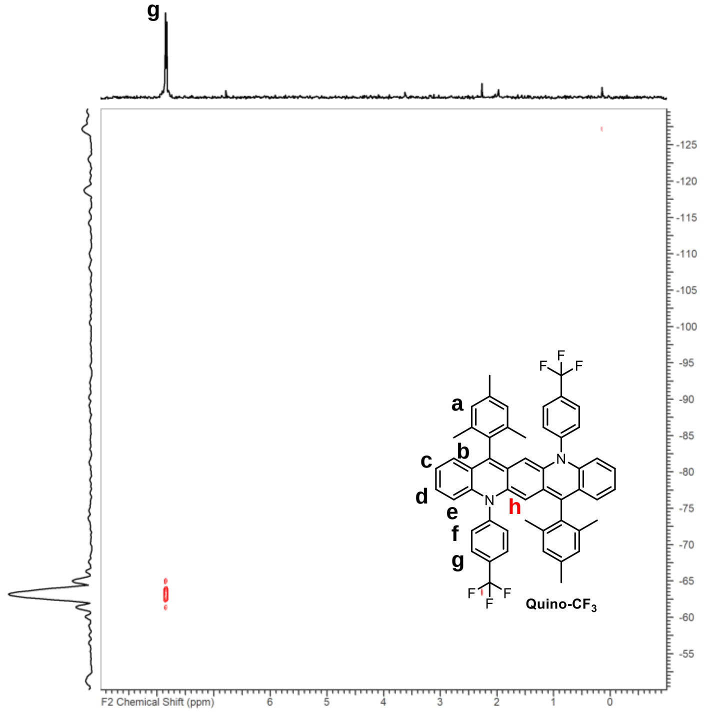

^{1}H, ^{19}F COSY

Acquisition Time (sec)	(0.2135, 0.0057)	*Comment*	Graf A_19F_hetCOSY THF /opt/topspin oci 42
Date	22 May 2019 09:57:36		
File Name	\\bunz22\Mitarbeiter\Gaozhan XIE\03_analytics\01-NMR\nmr02\c190528ubgz.388\40022\PDATA\1\2rr		
Frequency (MHz)	(399.8900, 376.2726)	*Nucleus*	(1H, 19F)
Number of Transients	8	*Origin*	spect
Original Points Count	(1024, 128)	*Owner*	auto
Points Count	(2048, 1024)	*Pulse Sequence*	cosygphfqfqn.jg
Solvent	THF	*Spectrum Type*	COSY
Sweep Width (Hz)	(4793.06, 22602.34)	*Temperature (degree C)*	22.148
Title	Graf A_19F_hetCOSY THF /opt/topspin oci 42		

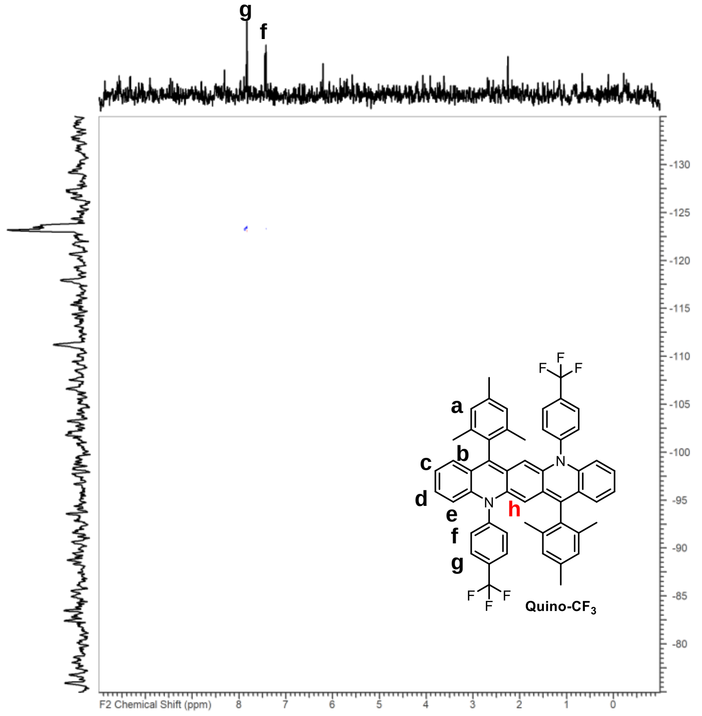

Quino-tBu^{2+}

^{1}H NMR

Multiplets Integrals Sum	57.91	Number of Nuclei	58 H's

Acquisition Time (sec)	3.6351	Comment	GZ 389	D	0.1	D1	0.1	DE	12
DS	2	Date	15 Apr 2019 02:20:46			Date Stamp	15 Apr 2019 02:20:46		
File Name	\\bunz22\Mitarbeiter\Gaozhan XIE\03_analytics\01-NMR\nmr02\e190412ubgz.389\2\PDATA\1\1r					Frequency (MHz)	600.2438	GB	0
INSTRUM	<spect>	LB	0.3	NS	128	Nucleus	1H	Number of Transients	128
Origin	spect	Original Points Count	65536	Owner	ns	PC	1		
PROBHD	<Z132808_0001 (CP QCI 600S3 H/P/C-N-D-05 Z LT)>					PULPROG	<zg30>	Points Count	65536
Pulse Sequence	zg30	Receiver Gain	15.35	SF	600.243829	SFO1	600.246830219145		
SI	65536	SSB	0	SW(cyclical) (Hz)	18028.85	SWH	18028.8461538462		
Solvent	ACETONITRILE-d3			Spectrum Offset (Hz)	2812.9792	Spectrum Type	standard	Sweep Width (Hz)	18028.57
TD	131072	TD0	16	TE	294.9998	Temperature (degree C)	22.000	UNC1	<1H>
WDW	1								

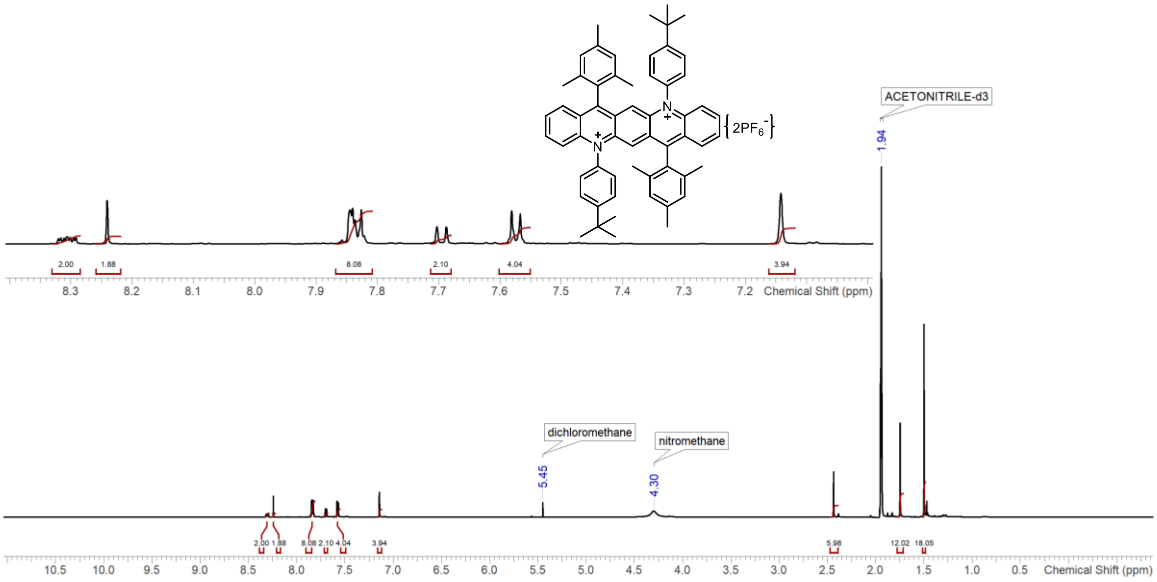

^{13}C {^{1}H} NMR

Multiplets Integrals Sum	0.00	Number of Nuclei	0 C's

Acquisition Time (sec)	1.0795	Comment	GZ 389	D	0.03	D1	2	DE	18
DS	4	Date	15 Apr 2019 02:11:46			Date Stamp	15 Apr 2019 02:11:46		
File Name	\\bunz22\Mitarbeiter\Gaozhan XIE\03_analytics\01-NMR\nmr02\e190412ubgz.389\1\PDATA\1\1r					Frequency (MHz)	150.9314	GB	0
INSTRUM	<spect>	LB	1	NS	4096	Nucleus	13C	Number of Transients	4096
Origin	spect	Original Points Count	49066	Owner	ns	PC	1.4		
PROBHD	<Z132808_0001 (CP QCI 600S3 H/P/C-N-D-05 Z LT)>			PULPROG	<zgpg30>	Points Count	65536	Pulse Sequence	zgpg30
Receiver Gain	2050.00	SF	150.931431	SFO1	150.94803345741			SI	65536
SSB	0	SW(cyclical) (Hz)	45454.55	SWH	45454.5454545455			Solvent	ACETONITRILE-d3
Spectrum Offset (Hz)	16718.0684	Spectrum Type	standard	Sweep Width (Hz)	45453.85	TD	98132	TD0	512
TE	294.9991	Temperature (degree C)	21.999	UNC1	<13C>	WDW	1		

User Notes	GZ 389

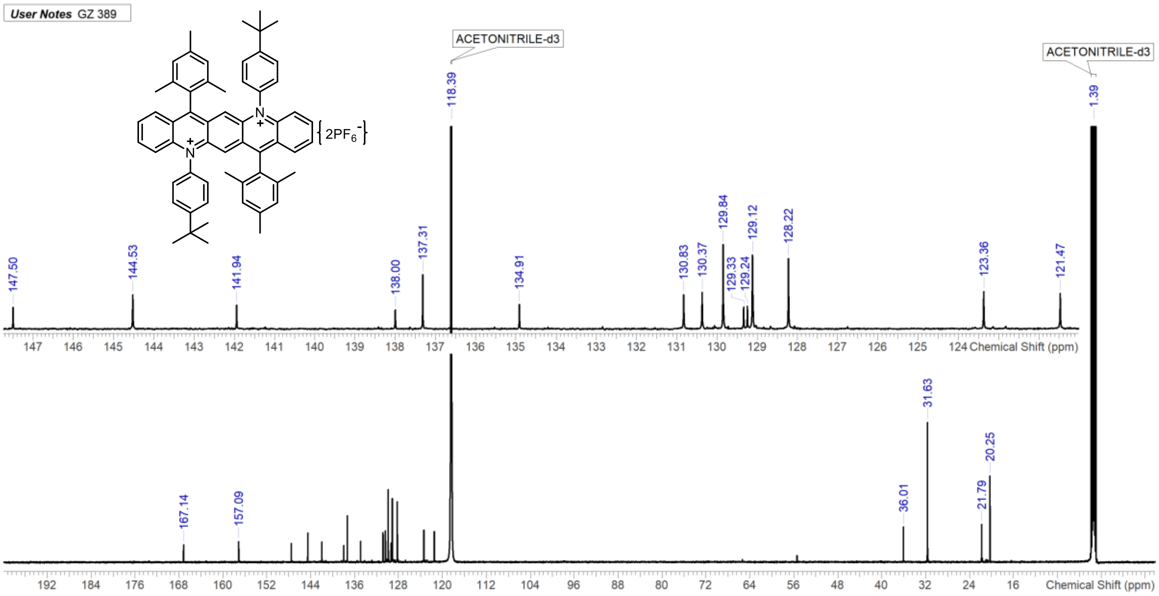

Quino-CF_3^{2+}

^1H NMR

Multiplets Integrals Sum	39.97	Number of Nuclei	40 H's

Acquisition Time (sec)	3.6351	Comment	GZ 397	D	0.1	D1	0.1	DE	12
DS	2	Date	18 Apr 2019 13:21:21			Date Stamp	18 Apr 2019 13:21:21		
File Name	\\bunz22\Mitarbeiter\Gaozhan XIE\03_analytics\01-NMR\nmr02\e190418ubgz.397\2\PDATA\1\1r					Frequency (MHz)	600.2438	GB	0
INSTRUM	<spect>	LB	0.3	NS	128	Nucleus	1H	Number of Transients	128
Origin	spect	Original Points Count	65536	Owner	ns	PC	1		
PROBHD	<Z132808_0001 (CP QCI 600S3 H/P/C-N-D-05 Z LT)>					PULPROG	<zg30>	Points Count	65536
Pulse Sequence	zg30	Receiver Gain	15.35	SF	600.243829	SFO1	600.246830219145		
SI	65536	SSB	0	SW(cyclical) (Hz)	18028.85	SWH	18028.8461538462		
Solvent	ACETONITRILE-d3			Spectrum Offset (Hz)	2813.2793	Spectrum Type	standard	Sweep Width (Hz)	18028.57
TD	131072	TD0	16	TE	295.0009	Temperature (degree C)	22.001	UNC1	<1H>
WDW	1								

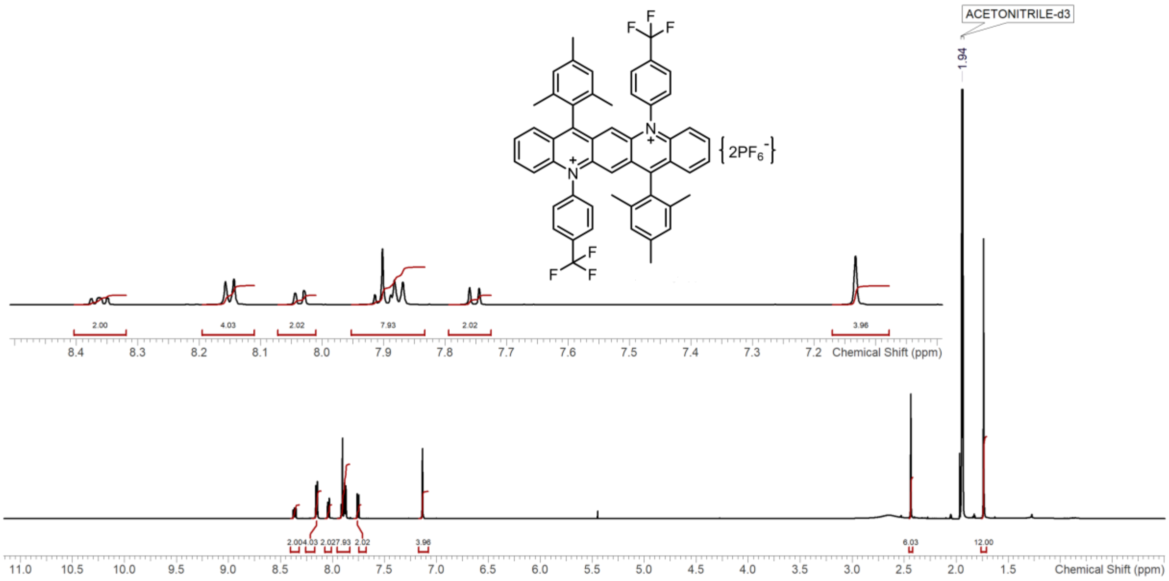

^{13}C {^1H} NMR

Multiplets Integrals Sum	0.00	Number of Nuclei	0 C's

Acquisition Time (sec)	1.0795	Comment	GZ 397	D	0.03	D1	2	DE	18
DS	4	Date	18 Apr 2019 13:12:19			Date Stamp	18 Apr 2019 13:12:19		
File Name	\\bunz22\Mitarbeiter\Gaozhan XIE\03_analytics\01-NMR\nmr02\e190418ubgz.397\1\PDATA\1\1r					Frequency (MHz)	150.9314	GB	0
INSTRUM	<spect>	LB	1	NS	4096	Nucleus	13C	Number of Transients	4096
Origin	spect	Original Points Count	49066	Owner	ns	PC	1.4		
PROBHD	<Z132808_0001 (CP QCI 600S3 H/P/C-N-D-05 Z LT)>			PULPROG	<zgpg30>	Points Count	65536	Pulse Sequence	zgpg30
Receiver Gain	2050.00	SF	150.931431	SFO1	150.94803345741			SI	65536
SSB	0	SW(cyclical) (Hz)	45454.55	SWH	45454.5454545455			Solvent	ACETONITRILE-d3
Spectrum Offset (Hz)	16763.7422	Spectrum Type	standard	Sweep Width (Hz)	45453.85	TD	98132	TD0	512
TE	294.9997	Temperature (degree C)	22.000	UNC1	<13C>	WDW	1		

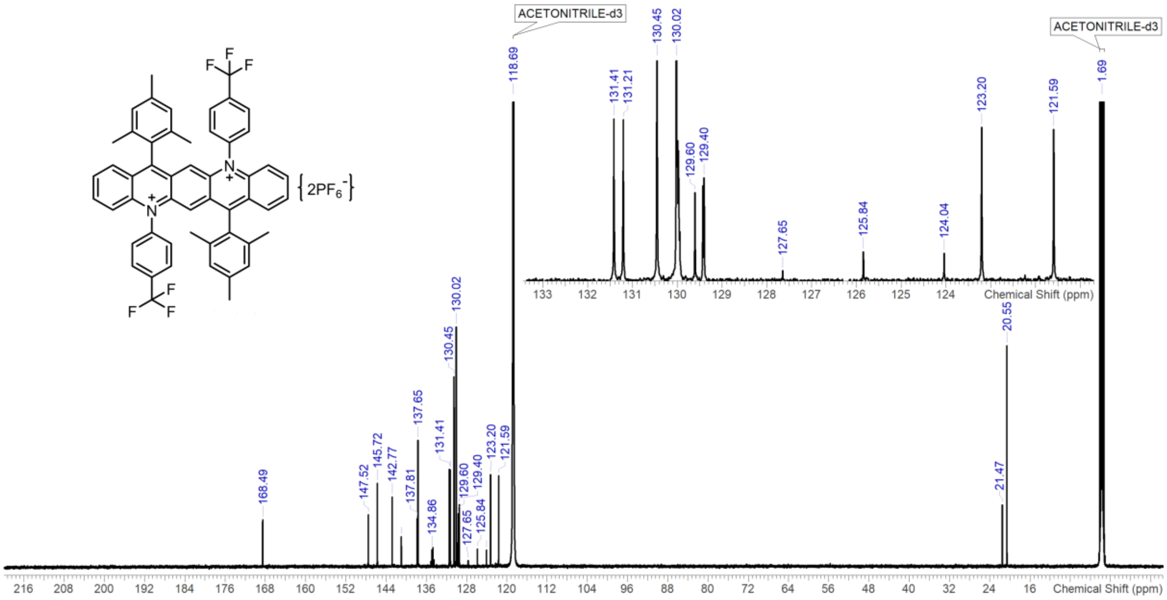

^{1}H, ^{1}H COSY

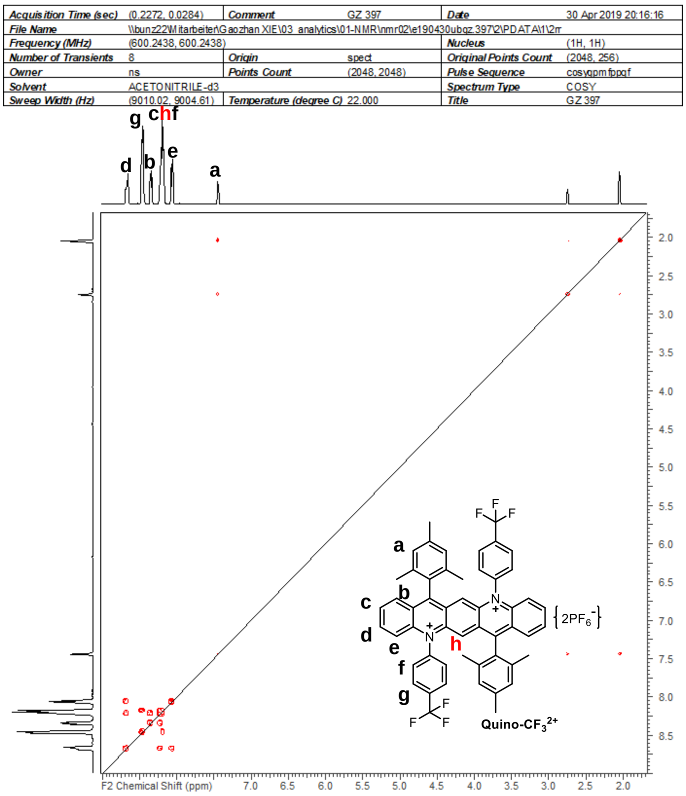

^{1}H, ^{1}H ROESY

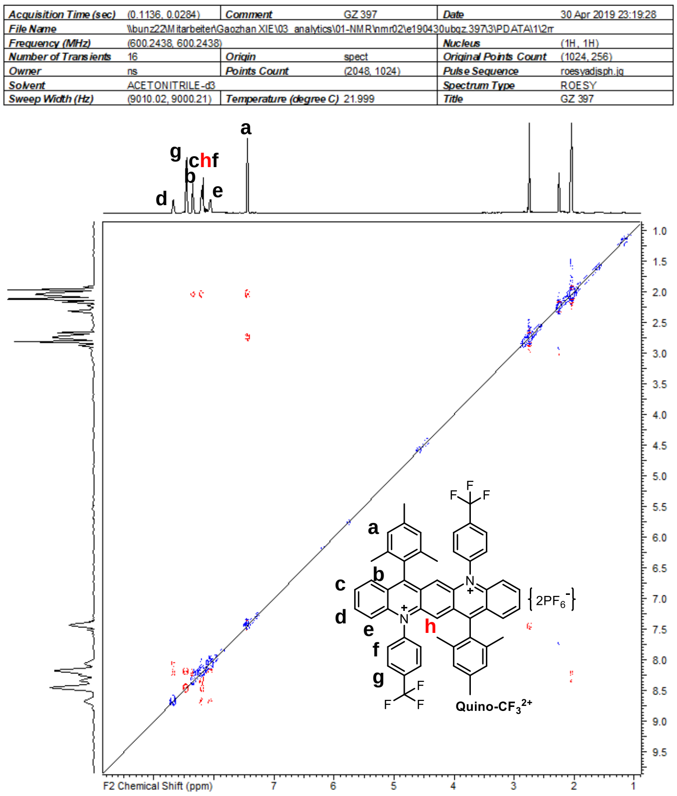

^{1}H, ^{15}N HMBC

Acquisition Time (sec)	(0.1704, 0.0052)	***Comment***	GZ 397	***Constant (Hz)***	8.0
Date	07 May 2019 23:07:22				
File Name	\\bunz22\Mitarbeiter\Gaozhan XIE\03_analytics\01-NMR\nmr02\c190507ubgz.397\2\PDATA\1\2rr				
Frequency (MHz)	(400.3300, 40.5651)			***Nucleus***	(1H, 15N)
Number of Transients	128	***Origin***	spect	***Original Points Count***	(1024, 128)
Owner	ns	***Points Count***	(2048, 2048)	***Pulse Sequence***	hmbcgpndqf
Solvent	ACETONITRILE-d3			***Spectrum Type***	HMBC
Sweep Width (Hz)	(6006.68, 24378.33)			***Temperature (degree C)***	21.999
Title	GZ 397				

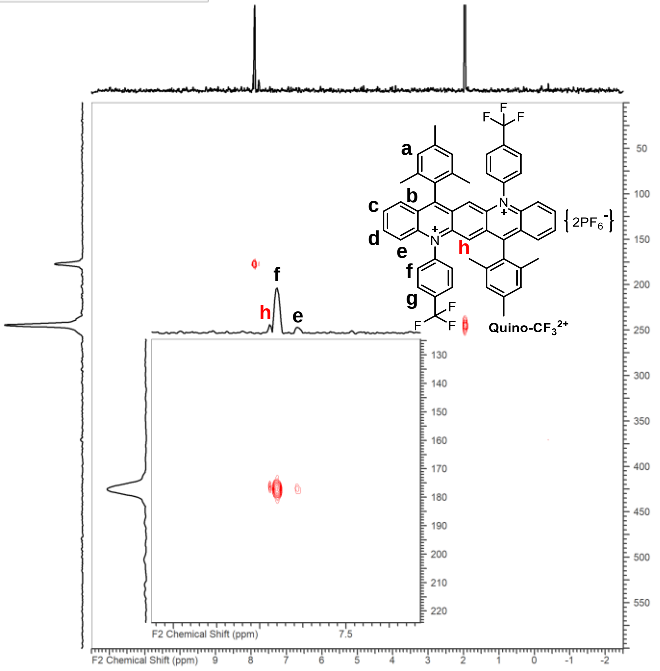

S7. Mass Spectrometry

N,N'-Bis(4-tert-butylphenyl)quinacridone (**QA-tBu**)

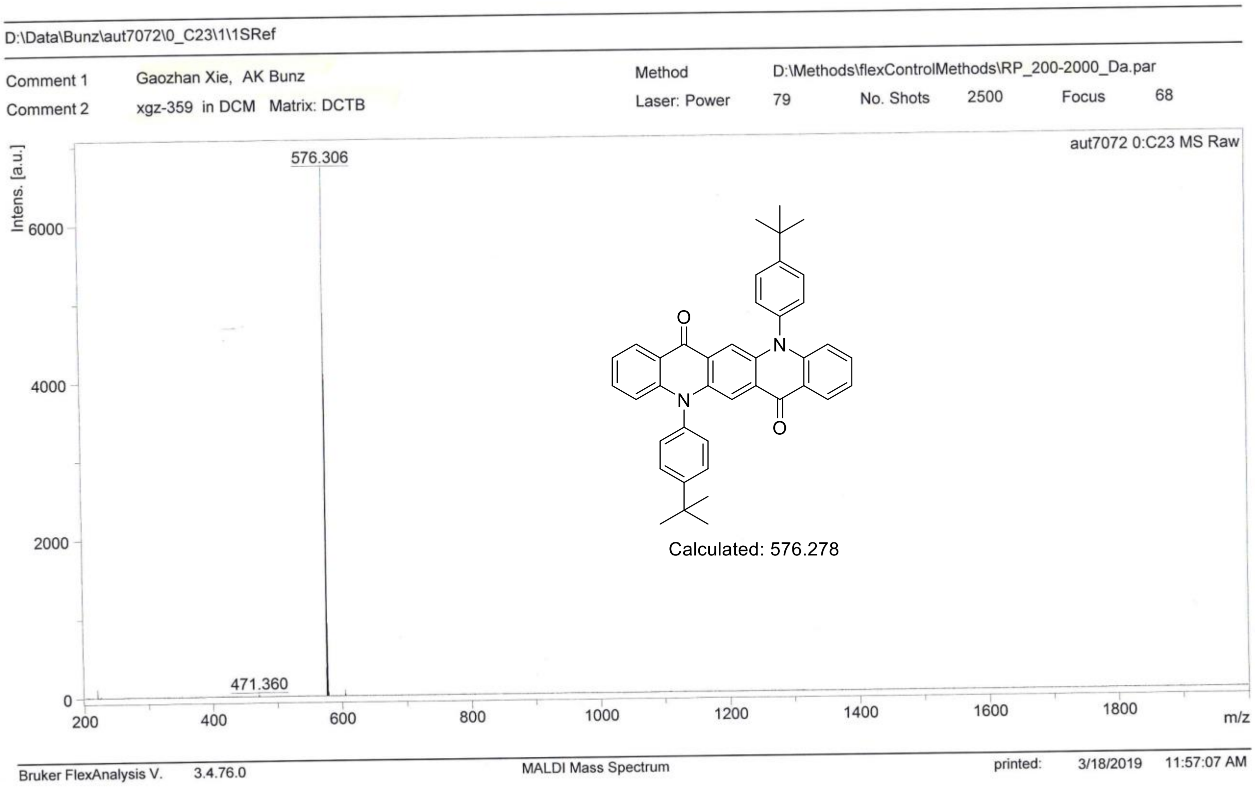

N,N'-Bis(4-methoxylphenyl)quinacridone (**QA-OMe**)

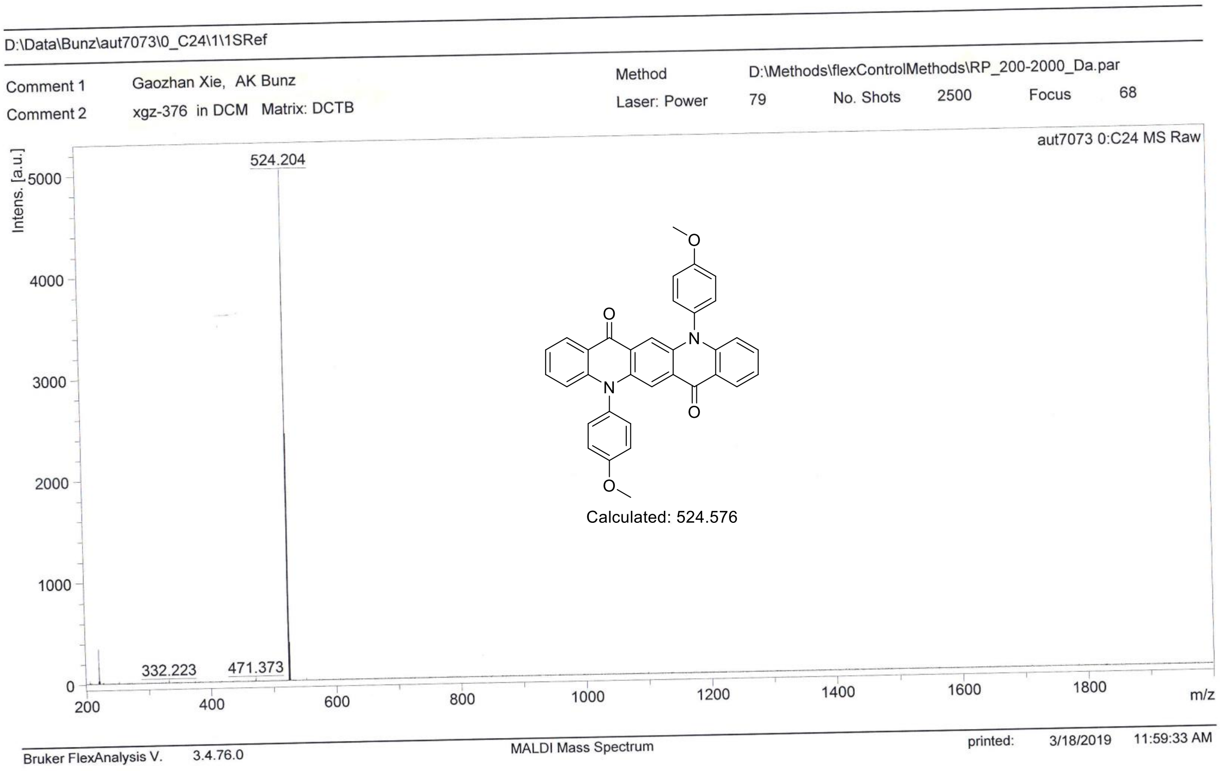

N,N'-Bis(4-trifluoromethylphenyl)quinacridone (**QA-CF$_3$**)

Analysis Info

Analysis Name	D:\Data\Bunz\icr33989_0_A14_000001.d	Acquisition Date	4/1/2019 1:17:17 PM
Method	MALDIposCsI_300-2500_512k	Instrument	ICR Apex-Qe
Sample Name	xgz-386	Operator	D.Lang
Comment	Gaozhan Xie, AK Bunz: xgz-386 in DCM Matrix: DCTB		

Acquisition Parameters

Accumulations	24	Collision Gas Flow Rate	0.8 L/sec	Laser Power	23.0 %
Broadband Low Mass	288.7 m/z	Collision Energy	0.5 eV	MALDI Plate	350.0 V
Broadband High Mass	2500.0 m/z	Collision Cell RF	1800.0 V	Calibration Date	Tue Mar 19 02:29:19 2019
Data Acquisition Size	524288	Q1 Resolution	10.0		
		Q1 Mass	200.000 m/z		

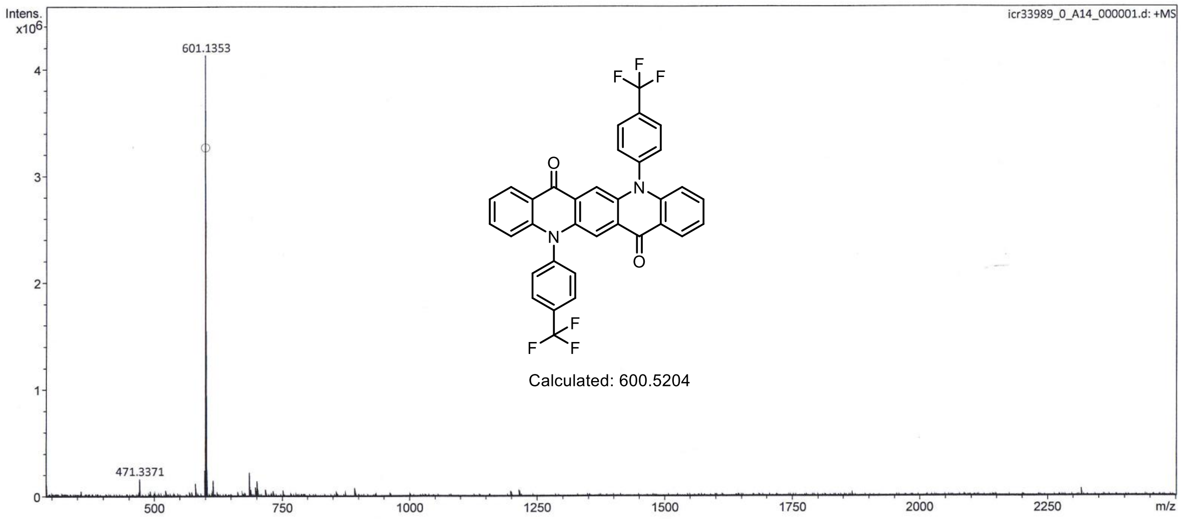

Spectrum Display Report Bruker Compass DataAnalysis 4.3 printed: 4/1/2019 1:18:48 PM Page 1 of 1

7,14-Dimesityl-5,12-dihydro-5,12-bis(4-tert-butylphenyl)diazapentacene (**Quino-tBu**)

Analysis Info

Analysis Name	D:\Data\Bunz\icr33555_0_I20_000002.d	Acquisition Date	2/21/2019 8:58:31 AM
Method	MALDIposCsI_300-2500_512k	Instrument	ICR Apex-Qe
Sample Name	xgz-369	Operator	D.Lang
Comment	Gaozhan Xie, AK Bunz: xgz-369 in DCM Matrix: DCTB		

Acquisition Parameters

Accumulations	16	Collision Gas Flow Rate	0.8 L/sec	Laser Power	23.0 %
Broadband Low Mass	288.7 m/z	Collision Energy	0.5 eV	MALDI Plate	350.0 V
Broadband High Mass	2500.0 m/z	Collision Cell RF	1800.0 V	Calibration Date	Thu Jan 17 09:47:46 2019
Data Acquisition Size	524288	Q1 Resolution	10.0		
		Q1 Mass	200.000 m/z		

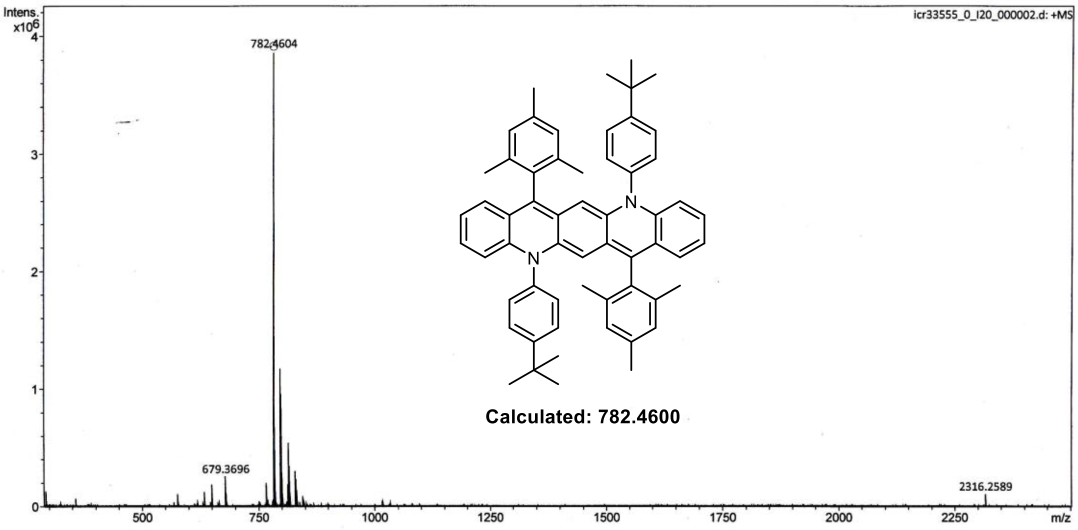

Spectrum Display Report Bruker Compass DataAnalysis 4.3 printed: 2/21/2019 8:59:50 AM Page 1 of 1

7,14-Dimesityl-5,12-dihydro-5,12-bis(4-methoxylphenyl)diazapentacene (**Quino-OMe**)

Analysis Info

Analysis Name	D:\Data\Bunz\icr34034_0_C14_000002.d	Acquisition Date	4/4/2019 9:17:09 AM
Method	MALDIposCsI_300-2500_512k	Instrument	ICR Apex-Qe
Sample Name	xgz-378	Operator	D.Lang
Comment	Gaozhan Xie, AK Bunz: xgz-378 in DCM Matrix: DCTB		

Acquisition Parameters

Accumulations	24	Collision Gas Flow Rate	0.8 L/sec	Laser Power	23.0 %
Broadband Low Mass	288.7 m/z	Collision Energy	0.5 eV	MALDI Plate	350.0 V
Broadband High Mass	2500.0 m/z	Collision Cell RF	1800.0 V	Calibration Date	Tue Mar 19 02:29:19 2019
Data Acquisition Size	524288	Q1 Resolution	10.0		
		Q1 Mass	200.000 m/z		

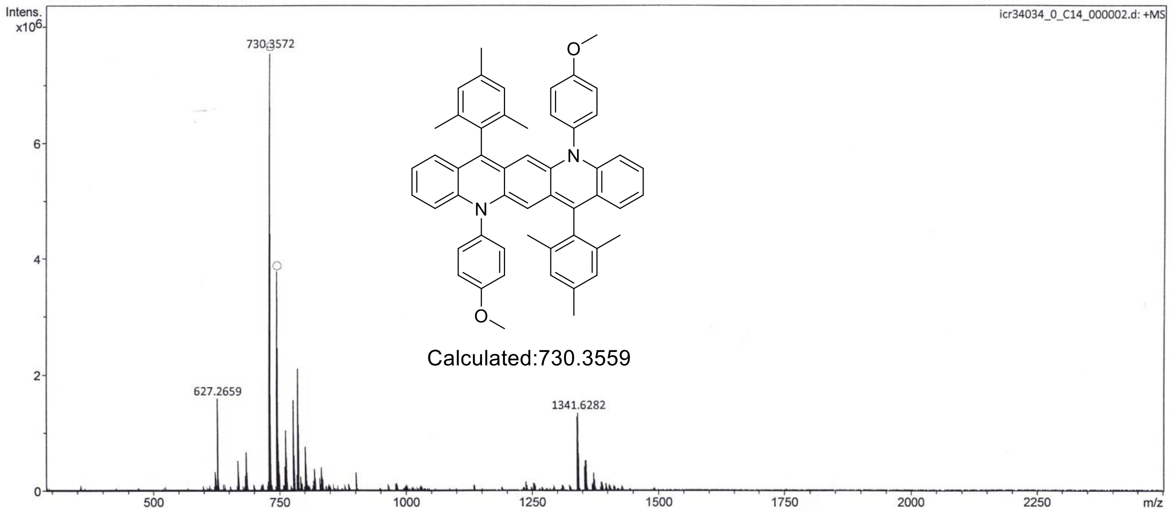

Spectrum Display Report Bruker Compass DataAnalysis 4.3 printed: 4/4/2019 9:18:36 AM Page 1 of 1

7,14-Dimesityl-5,12-dihydro-5,12-bis(4-trifluoromethylphenyl)diazapentacene (**Quino-CF_3**)

Analysis Info

Analysis Name	Z:\Bunz\ic33917_0_L24_000001.d	Acquisition Date	27.03.2019 10:41:31
Method	MALDIposCsI_300-2500_512k	Instrument	ICR Apex-Qe
Sample Name		Operator	D.Lang
Comment	Gaozhan Xie, AK Bunz: xgz-388 in DCM Matrix: DCTB		

Acquisition Parameters

Accumulations	24	Collision Gas Flow Rate	0.8 L/sec	Laser Power	23.0 %
Broadband Low Mass	288.7 m/z	Collision Energy	0.5 eV	MALDI Plate	350.0 V
Broadband High Mass	2500.0 m/z	Collision Cell RF	1800.0 V	Calibration Date	Tue Mar 19 02:29:19 2019
Data Acquisition Size	524288	Q1 Resolution	10.0		
		Q1 Mass	200.000 m/z		

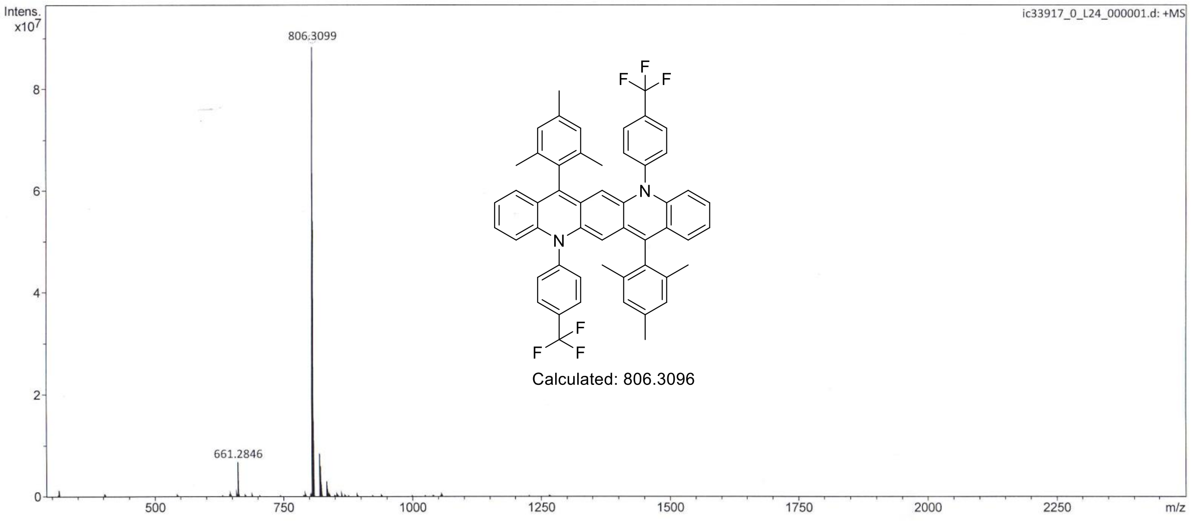

Spectrum Display Report Bruker Compass DataAnalysis 4.3 printed: 27.03.2019 11:09:30 Page 1 of 1

S8. Infrared Spectroscopy

N,N'-Bis(4-tert-butylphenyl)quinacridone (**QA-tBu**)

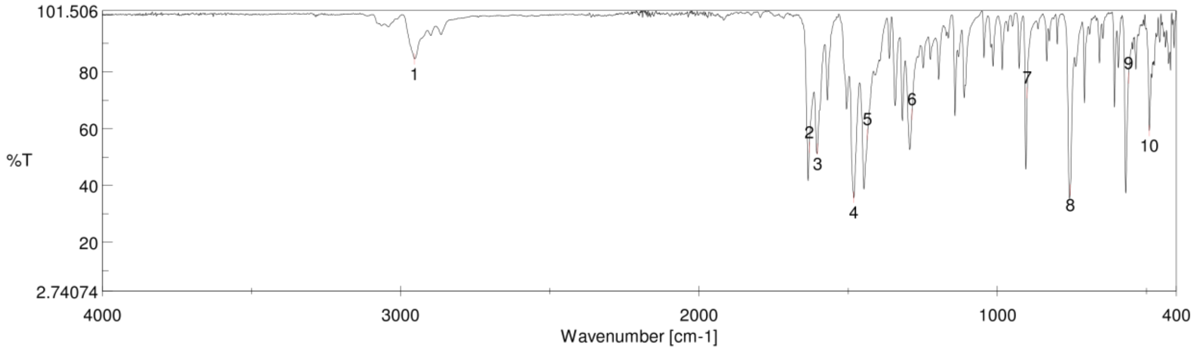

[Result of Peak Picking]

No.	Position	Intensity	No.	Position	Intensity	No.	Position	Intensity
1	2953.45	84.5058	2	1630.04	53.3593	3	1602.56	52.784
4	1481.54	35.7649	5	1434.78	57.9652	6	1286.29	64.9186
7	899.63	72.5863	8	755.477	38.3379	9	560.22	77.7554
10	490.313	59.2333						

N,N'-Bis(4-methoxylphenyl)quinacridone (**QA-OMe**)

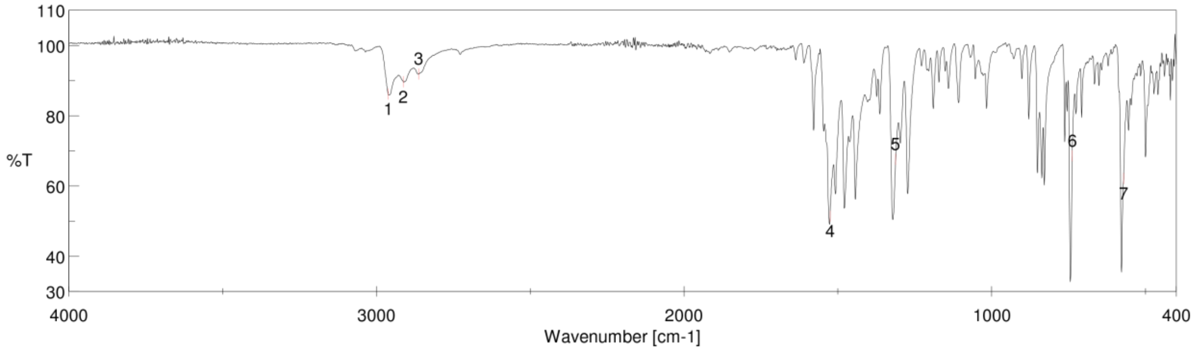

[Result of Peak Picking]

No.	Position	Intensity	No.	Position	Intensity	No.	Position	Intensity
1	2962.13	86.1473	2	2912.95	89.6512	3	2863.29	91.8477
4	1525.42	51.3721	5	1312.32	67.5647	6	738.121	68.5308
7	571.308	62.1011						

N,N'-Bis(4-trifluoromethylphenyl)quinacridone (**QA-CF_3**)

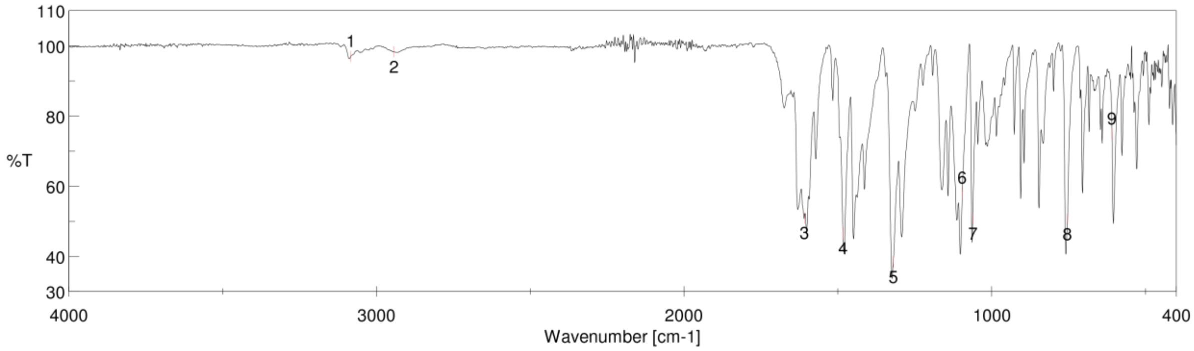

[Result of Peak Picking]

No.	Position	Intensity	No.	Position	Intensity	No.	Position	Intensity
1	3083.62	96.8944	2	2943.32	98.2481	3	1608.82	50.9172
4	1483.47	46.5841	5	1320.04	38.3994	6	1095.85	58.0533
7	1061.62	50.6841	8	753.548	50.6357	9	608.913	74.8735

7,14-Dimesityl-5,12-dihydro-5,12-bis(4-tert-butylphenyl)diazapentacene (**Quino-tBu**)

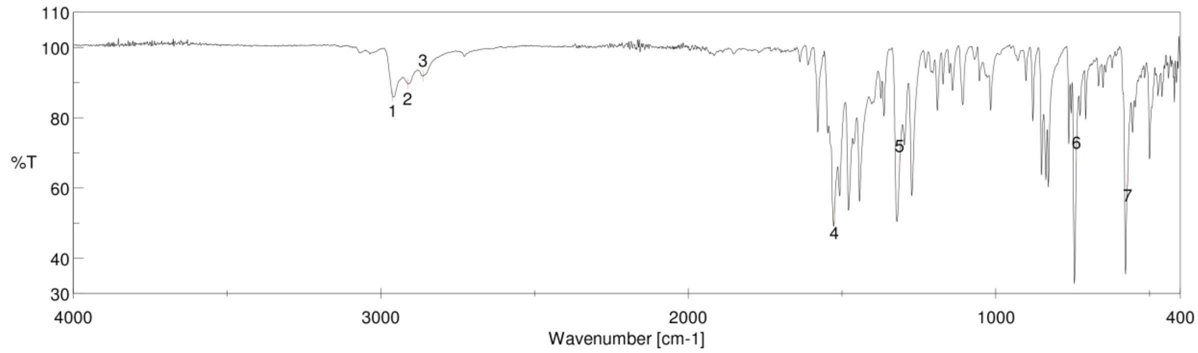

[Result of Peak Picking]

No.	Position	Intensity	No.	Position	Intensity	No.	Position	Intensity
1	2962.13	86.1473	2	2912.95	89.6512	3	2863.29	91.8477
4	1525.42	51.3721	5	1312.32	67.5647	6	738.121	68.5308
7	571.308	62.1011						

7,14-Dimesityl-5,12-dihydro-5,12-bis(4-methoxylphenyl)diazapentacene (**Quino-OMe**)

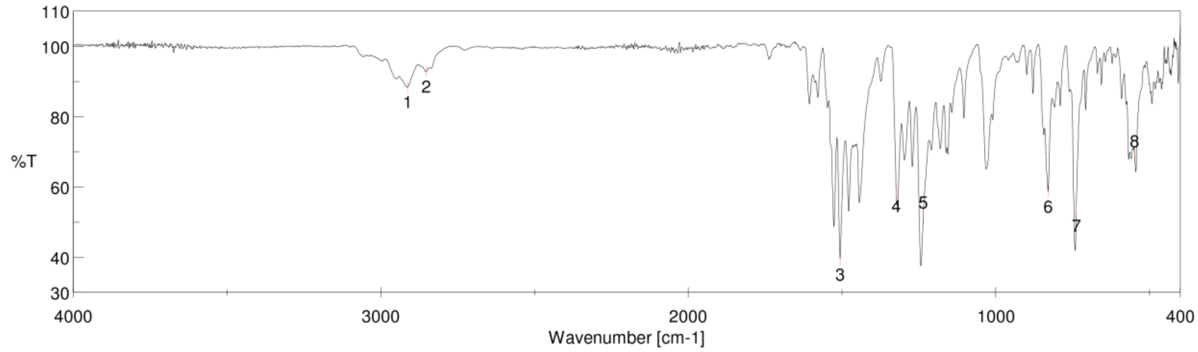

[Result of Peak Picking]

No.	Position	Intensity	No.	Position	Intensity	No.	Position	Intensity
1	2912.95	88.3776	2	2851.72	92.816	3	1506.13	39.3606
4	1323.89	58.8554	5	1236.15	51.2263	6	829.723	58.7306
7	738.121	53.2082	8	548.167	68.6837			

7,14-Dimesityl-5,12-dihydro-5,12-bis(4-trifluoromethylphenyl)diazapentacene (**Quino-CF_3**)

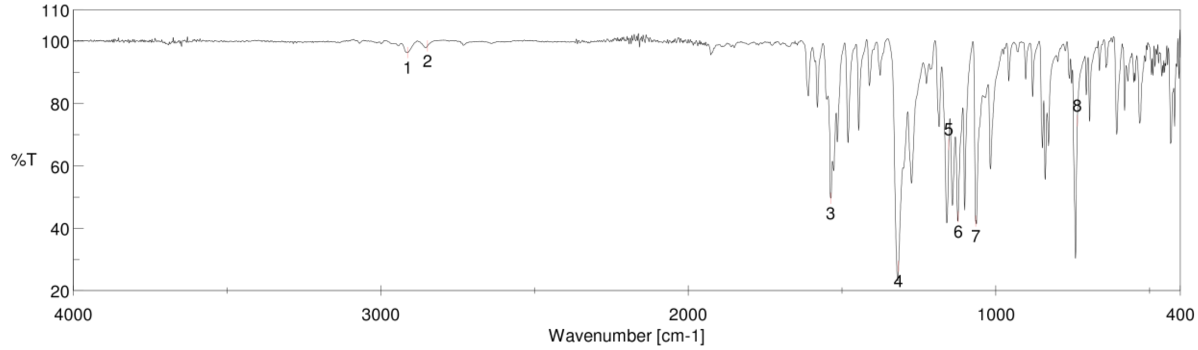

[Result of Peak Picking]

No.	Position	Intensity	No.	Position	Intensity	No.	Position	Intensity
1	2912.95	96.2619	2	2848.35	98.4038	3	1536.51	49.6059
4	1316.18	27.814	5	1152.74	66.7948	6	1122.37	43.6522
7	1065.48	42.233	8	734.746	74.3326			

Quino-tBu$^{+\bullet}$

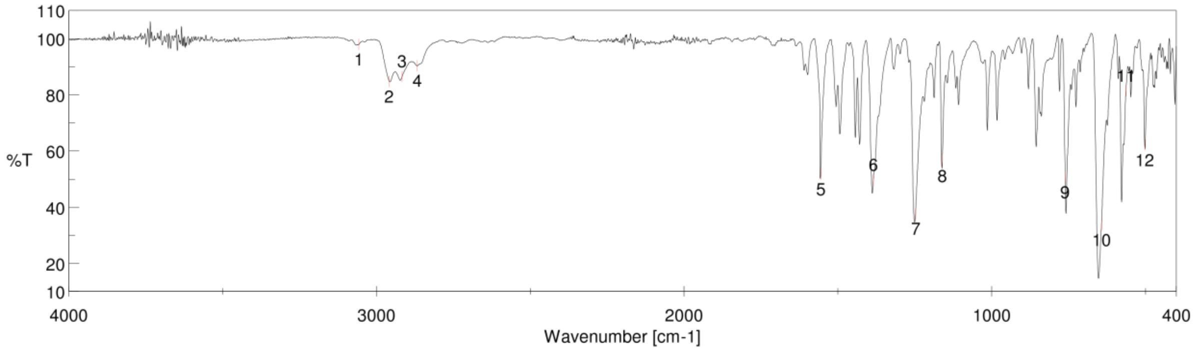

[Result of Peak Picking]

No.	Position	Intensity	No.	Position	Intensity	No.	Position	Intensity
1	3057.1	97.932	2	2958.27	84.7808	3	2916.81	86.4873
4	2867.15	90.2868	5	1555.79	51.7605	6	1384.64	49.6529
7	1247.72	37.638	8	1160.45	56.4792	9	761.262	50.6062
10	643.144	33.6831	11	563.594	81.3019	12	502.848	62.1381

Quino-CF$_3^{+\bullet}$

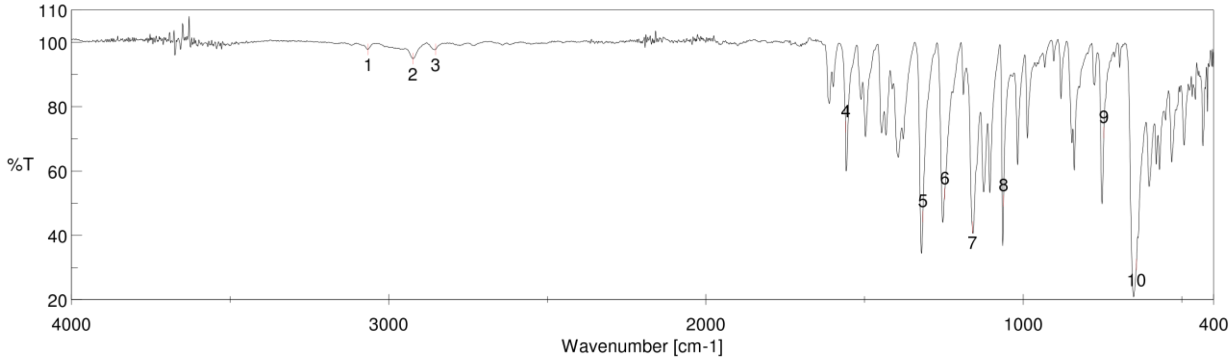

[Result of Peak Picking]

No.	Position	Intensity	No.	Position	Intensity	No.	Position	Intensity
1	3064.82	97.7568	2	2924.04	94.869	3	2851.72	97.77
4	1559.65	73.6753	5	1316.18	45.7554	6	1247.72	52.8973
7	1160.45	42.4512	8	1061.62	50.7301	9	745.835	71.8619
10	643.144	30.8652						

Quino-tBu^{2+}

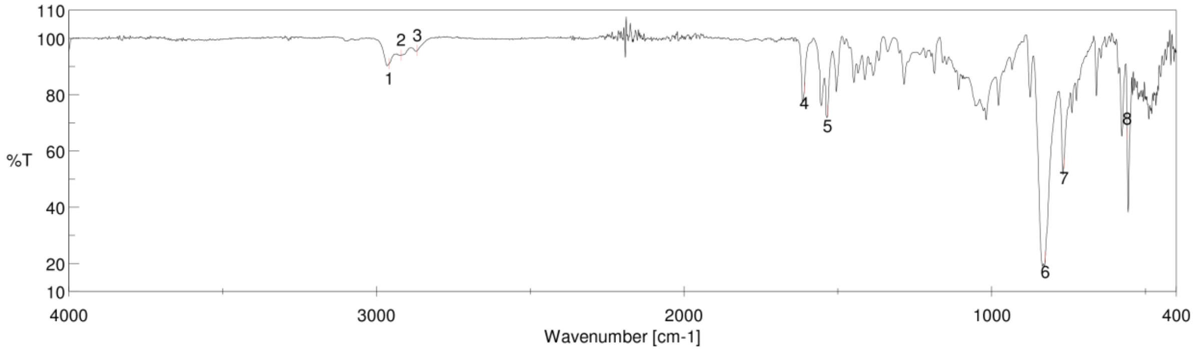

[Result of Peak Picking]

No.	Position	Intensity	No.	Position	Intensity	No.	Position	Intensity
1	2958.27	91.0033	2	2920.18	94.0412	3	2867.15	95.6683
4	1608.82	82.3424	5	1532.65	74.1405	6	825.866	22.1889
7	765.119	55.6086	8	559.738	65.9708			

Quino-CF_3^{2+}

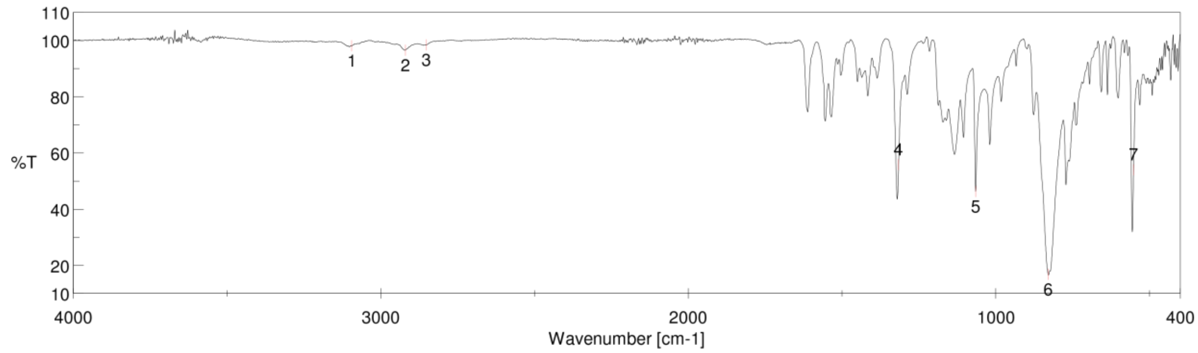

[Result of Peak Picking]

No.	Position	Intensity	No.	Position	Intensity	No.	Position	Intensity
1	3095.19	98.0837	2	2920.18	96.5435	3	2851.72	98.3761
4	1316.18	55.786	5	1065.48	46.2623	6	829.723	16.7884
7	552.024	53.9361						

S9. Crystals Structures

Quino-tBu

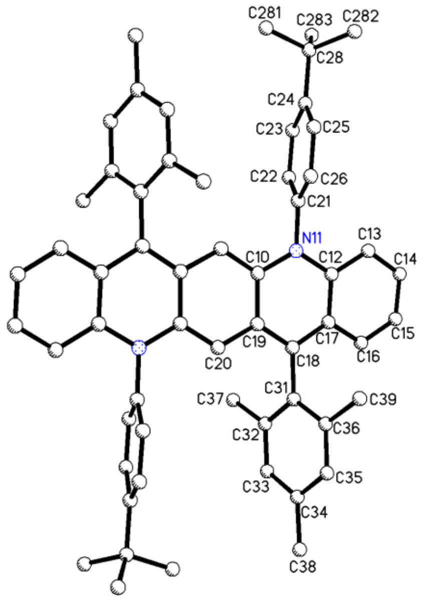

Identification code	gxi14	
Empirical formula	$C_{58}H_{58}N_2$	
Formula weight	783.06	
Temperature	200(2) K	
Wavelength	1.54178 Å	
Crystal system	monoclinic	
Space group	$P2_1/n$	
Z	2	
Unit cell dimensions	a =9.4682(3) Å	α = 90 deg.
	b = 13.7037(3) Å	β =97.603(3) deg.
	c = 17.4850(6) Å	γ = 90 deg.
Volume	2248.72(12) Å^3	
Density (calculated)	1.16 g/cm^3	
Absorption coefficient	0.50 mm^{-1}	
Crystal shape	plank	
Crystal size	0.076 x 0.075 x 0.045 mm^3	
Crystal colour	purple	

Theta range for data collection	5.1 to 72.1 deg.
Index ranges	-11≤h≤10, -16≤k≤7, -19≤l≤21
Reflections collected	15084
Independent reflections	4319 (R(int) = 0.0229)
Observed reflections	3192 (I > 2σ(I))
Absorption correction	Semi-empirical from equivalents
Max. and min. transmission	1.33 and 0.69
Refinement method	Full-matrix least-squares on F^2
Data/restraints/parameters	4319 / 0 / 277
Goodness-of-fit on F^2	1.03
Final R indices (I>2sigma(I))	R1 = 0.049, wR2 = 0.118
Largest diff. peak and hole	0.26 and -0.19 eÅ^{-3}

Quino-CF_3

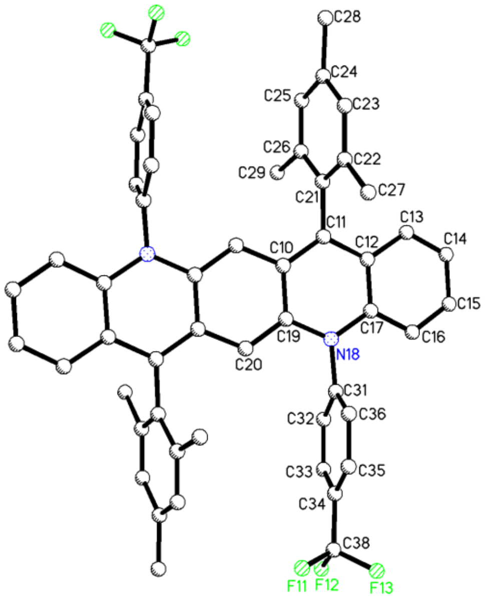

	Identification code	gxi12
Empirical formula	$C_{52}H_{40}F_6N_2$	
Formula weight	806.86	
Temperature	200(2) K	
Wavelength	1.54178 Å	
Crystal system	triclinic	
Space group	$P\bar{1}$	
Z	2	
Unit cell dimensions	a = 12.8417(4) Å	α =78.588(3) deg.
	b = 13.0652(4) Å	β =67.275(2) deg.
	c = 15.1490(5) Å	γ =61.268(2) deg.
Volume	2055.50(12) Å^3	
Density (calculated)	1.30 g/cm^3	
Absorption coefficient	0.78 mm^{-1}	
Crystal shape	plank	
Crystal size	0.219 x 0.048 x 0.028 mm^3	
Crystal colour	red	
Theta range for data collection	3.2 to 69.6 deg.	
Index ranges	-15≤h≤15, -15≤k≤13, -16≤l≤18	
Reflections collected	19919	
Independent reflections	7408 (R(int) = 0.0194)	
Observed reflections	5644 (I > 2σ(I))	
Absorption correction	Semi-empirical from equivalents	

Max. and min. transmission	1.43 and 0.64
Refinement method	Full-matrix least-squares on F^2
Data/restraints/parameters	7408 / 297 / 573
Goodness-of-fit on F^2	1.04
Final R indices (I>2sigma(I))	R1 = 0.049, wR2 = 0.117
Largest diff. peak and hole	0.24 and -0.30 eÅ^{-3}

Quino-$CF_3^{+\cdot}$

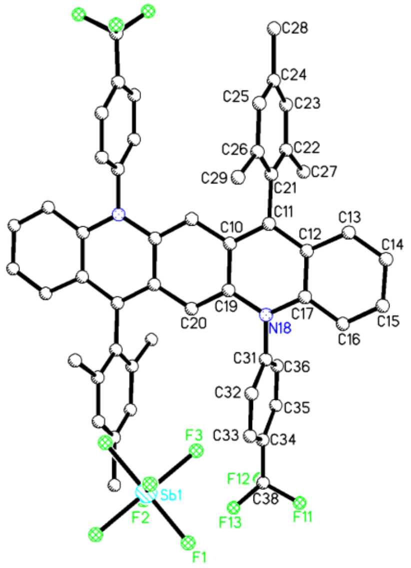

Identification code	gxi13	
Empirical formula	$C_{55}H_{46}F_{12}N_2OSb$	
Formula weight	1100.69	
Temperature	200(2) K	
Wavelength	0.71073 Å	
Crystal system	orthorhombic	
Space group	Pccn	
Z	4	
Unit cell dimensions	a = 20.8537(6) Å	α = 90 deg.
	b = 15.2043(4) Å	β = 90 deg.
	c = 15.7519(5) Å	γ = 90 deg.
Volume	4994.4(3) Å^3	
Density (calculated)	1.46 g/cm^3	
Absorption coefficient	0.64 mm^{-1}	
Crystal shape	plank	
Crystal size	0.159 x 0.070 x 0.026 mm^3	
Crystal colour	brown	
Theta range for data collection	1.7 to 25.1 deg.	
Index ranges	$-24 \leq h \leq 24$, $-18 \leq k \leq 18$, $-18 \leq l \leq 18$	
Reflections collected	58204	
Independent reflections	4427 (R(int) = 0.0926)	
Observed reflections	2585 ($I > 2\sigma(I)$)	
Absorption correction	Semi-empirical from equivalents	
Max. and min. transmission	0.96 and 0.88	
Refinement method	Full-matrix least-squares on F^2	
Data/restraints/parameters	4427 / 96 / 340	
Goodness-of-fit on F^2	1.03	
Final R indices (I>2sigma(I))	R1 = 0.055, wR2 = 0.143	
Largest diff. peak and hole	0.70 and -0.55 eÅ^{-3}	

S10. References

[1] Cardona, C. M.; Li, W.; Kaifer, A. E.; Stockdale, D.; Bazan, G. C.; *Adv. Mater.* **2011**, 23, 2367-2371.
